# Genome-Wide Association Study of White Blood Cell Count in 16,388 African Americans: the Continental Origins and Genetic Epidemiology Network (COGENT)

**DOI:** 10.1371/journal.pgen.1002108

**Published:** 2011-06-30

**Authors:** Alexander P. Reiner, Guillaume Lettre, Michael A. Nalls, Santhi K. Ganesh, Rasika Mathias, Melissa A. Austin, Eric Dean, Sampath Arepalli, Angela Britton, Zhao Chen, David Couper, J. David Curb, Charles B. Eaton, Myriam Fornage, Struan F. A. Grant, Tamara B. Harris, Dena Hernandez, Naoyuki Kamatini, Brendan J. Keating, Michiaki Kubo, Andrea LaCroix, Leslie A. Lange, Simin Liu, Kurt Lohman, Yan Meng, Emile R. Mohler, Solomon Musani, Yusuke Nakamura, Christopher J. O'Donnell, Yukinori Okada, Cameron D. Palmer, George J. Papanicolaou, Kushang V. Patel, Andrew B. Singleton, Atsushi Takahashi, Hua Tang, Herman A. Taylor, Kent Taylor, Cynthia Thomson, Lisa R. Yanek, Lingyao Yang, Elad Ziv, Alan B. Zonderman, Aaron R. Folsom, Michele K. Evans, Yongmei Liu, Diane M. Becker, Beverly M. Snively, James G. Wilson

**Affiliations:** 1 Department of Epidemiology, University of Washington, Seattle, Washington, United States of America; 2 Division of Public Health Sciences, Fred Hutchinson Cancer Research Center, Seattle, Washington, United States of America; 3 Montreal Heart Institute, Montréal, Canada; 4 Département de Médecine, Université de Montréal, Montréal, Canada; 5 Laboratory of Neurogenetics, National Institute on Aging, National Institutes of Health, Bethesda, Maryland, United States of America; 6 Division of Cardiovascular Medicine, Department of Internal Medicine, University of Michigan, Ann Arbor, Michigan, United States of America; 7 Department of Medicine, The Johns Hopkins University School of Medicine, Baltimore, Maryland, United States of America; 8 Department of Epidemiology and Institute for Public Health Genetics, School of Public Health, University of Washington, Seattle, Washington, United States of America; 9 Department of Medicine, University of California San Francisco, San Francisco, California, United States of America; 10 Division of Epidemiology and Biostatistics, Mel and Enid Zuckerman College of Public Health, University of Arizona, Tucson, Arizona, United States of America; 11 Department of Epidemiology, University of North Carolina School of Public Health, Chapel Hill, North Carolina, United States of America; 12 Department of Geriatric Medicine, John A. Burns School of Medicine, University of Hawaii, Honolulu, Hawaii, United States of America; 13 Center for Primary Care and Prevention, Alpert Medical School of Brown University, Providence, Rhode Island, United States of America; 14 Houston Institute of Molecular Medicine, University of Texas, Houston, Texas, United States of America; 15 Center for Applied Genomics, Division of Human Genetics, Children's Hospital of Philadelphia Research Institute, Philadelphia, Pennsylvania, United States of America; 16 Laboratory for Epidemiology, Demography, and Biometry, National Institute on Aging, National Institutes of Health, Baltimore, Maryland, United States of America; 17 Laboratory for Statistical Analysis, Center for Genomic Medicine (CGM), Institute of Physical and Chemical Research (RIKEN), Yokohama, Japan; 18 Laboratory for Genotyping Development, CGM, RIKEN, Yokohama, Japan; 19 Department of Genetics, University of North Carolina, Chapel Hill, North Carolina, United States of America; 20 Departments of Epidemiology and Medicine, University of California Los Angeles, Los Angeles, California, United States of America; 21 Center for Human Genomics, Department of Epidemiology and Prevention, Division of Public Health Sciences, Wake Forest University School of Medicine, Winston-Salem, North Carolina, United States of America; 22 Program in Medical and Population Genetics, Broad Institute, Cambridge, Massachusetts, United States of America; 23 Cardiovascular Division, Vascular Medicine Section, Department of Medicine, University of Pennsylvania School of Medicine, Philadelphia, Pennsylvania, United States of America; 24 Department of Medicine, University of Mississippi Medical Center, Jackson, Mississippi, United States of America; 25 Laboratory of Molecular Medicine, Human Genome Center, Institute of Medical Science, University of Tokyo, Tokyo, Japan; 26 National Heart, Lung, and Blood Institute (NHLBI), Division of Cardiovascular Sciences, Bethesda, Maryland, United States of America; 27 NHLBI's Framingham Heart Study, Framingham, Massachusetts, United States of America; 28 Department of Genetics, Stanford University School of Medicine, Stanford, California, United States of America; 29 Jackson State University, Tougaloo College, Jackson, Mississippi, United States of America; 30 Department of Medicine, University of Mississippi Medical Center, Jackson, Mississippi, United States of America; 31 Medical Genetics Institute, Cedars-Sinai Medical Center, Los Angeles, California, United States of America; 32 Nutritional Sciences, Arizona Cancer Center, University of Arizona, Tucson, Arizona, United States of America; 33 Department of Biostatistical Sciences, Division of Public Health Sciences, Wake Forest School of Medicine, Winston-Salem, North Carolina, United States of America; 34 Laboratory of Personality and Cognition, National Institute on Aging, National Institutes of Health, Baltimore, Maryland, United States of America; 35 Division of Epidemiology and Community Health, University of Minnesota, Minneapolis, Minnesota, United States of America; 36 Health Disparities Research Section, Clinical Research Branch, National Institute on Aging, National Institutes of Health, Baltimore, Maryland, United States of America; 37 Department of Physiology and Biophysics, University of Mississippi Medical Center, Jackson, Mississippi, United States of America; University of Michigan, United States of America

## Abstract

Total white blood cell (WBC) and neutrophil counts are lower among individuals of African descent due to the common African-derived “null” variant of the Duffy Antigen Receptor for Chemokines (*DARC*) gene. Additional common genetic polymorphisms were recently associated with total WBC and WBC sub-type levels in European and Japanese populations. No additional loci that account for WBC variability have been identified in African Americans. In order to address this, we performed a large genome-wide association study (GWAS) of total WBC and cell subtype counts in 16,388 African-American participants from 7 population-based cohorts available in the Continental Origins and Genetic Epidemiology Network. In addition to the *DARC* locus on chromosome 1q23, we identified two other regions (chromosomes 4q13 and 16q22) associated with WBC in African Americans (*P*<2.5×10^−8^). The lead SNP (rs9131) on chromosome 4q13 is located in the *CXCL2* gene, which encodes a chemotactic cytokine for polymorphonuclear leukocytes. Independent evidence of the novel *CXCL2* association with WBC was present in 3,551 Hispanic Americans, 14,767 Japanese, and 19,509 European Americans. The index SNP (rs12149261) on chromosome 16q22 associated with WBC count is located in a large inter-chromosomal segmental duplication encompassing part of the hydrocephalus inducing homolog (*HYDIN*) gene. We demonstrate that the chromosome 16q22 association finding is most likely due to a genotyping artifact as a consequence of sequence similarity between duplicated regions on chromosomes 16q22 and 1q21. Among the WBC loci recently identified in European or Japanese populations, replication was observed in our African-American meta-analysis for rs445 of *CDK6* on chromosome 7q21 and rs4065321 of *PSMD3-CSF3* region on chromosome 17q21. In summary, the *CXCL2*, *CDK6*, and *PSMD3-CSF3* regions are associated with WBC count in African American and other populations. We also demonstrate that large inter-chromosomal duplications can result in false positive associations in GWAS.

## Author Summary

Although recent genome-wide association studies have identified common genetic variants associated with total white blood cell (WBC) and WBC sub-type counts in European and Japanese ancestry populations, whether these or other loci account for differences in WBC count among African Americans is unknown. By examining >16,000 African Americans, we show that, in addition to the previously identified Duffy Antigen Receptor for Chemokines (*DARC*) locus on chromosome 1, another variant, rs9131, and other nearby variants on human chromosome 4 are associated with total WBC count in African Americans. The variants span the *CXCL2* gene, which encodes an inflammatory mediator involved in WBC production and migration. We show that the association is not restricted to African Americans but is also present in independent samples of European Americans, Hispanic Americans, and Japanese. This finding is potentially important because WBC mediate or have altered counts in a variety of acute and chronic disorders.

## Introduction

Proliferation and differentiation of hematopoietic stem cells into mature white blood cells (WBC) in the bone marrow, followed by release into the circulation of mature WBC, is a highly regulated process [1]. WBC comprise several subtypes including neutrophils, lymphocytes, monocytes, eosinophils, and basophils. These cells play an essential role in innate and adaptive immunity against invading microorganisms. They are also involved in the pathogenesis of various acute and chronic diseases. The circulating numbers of leukocytes can be influenced by stress, infection, or inflammation. Total WBC and neutrophil counts also differ by ethnicity, with levels 10–20% lower among African American than European American populations [2], [3]. This difference is due to a common African-derived “null” variant (rs2814778) of the Duffy Antigen Receptor for Chemokines (*DARC*) gene, which also confers selective advantage against malaria [4]–[6]. By abolishing expression of DARC on red blood cells, the Duffy null variant may alter the concentration and distribution of chemokines in the blood and tissue [7]–[10], thereby regulating neutrophil production and migration.

Several clinically distinct forms of congenital neutropenia are inherited as rare, monogenic disorders [11]. Genetic polymorphisms more common in the population, including those that reside in the region of 17q21 harboring the *CSF3* gene, were recently associated with circulating total WBC and WBC subtype counts in European and Japanese populations [12]–[15]. Yet these common polymorphisms account for only a fraction of the reported 50–60% heritability of WBC count [16]–[18]. In addition, the contribution of these or other loci to variation in total WBC or WBC subtypes have yet to be thoroughly evaluated through current genome-wide association approaches in other populations, such as African Americans. To identify additional polymorphisms associated with WBC and its subtypes (neutrophils, lymphocytes, monocytes, eosinophils, basophils), we therefore performed a large, multi-cohort genome wide association study (GWAS) of typed and imputed SNPs in African Americans, with follow-up in additional ethnic samples of European and Japanese ancestry.

## Results

**Figure 1:**
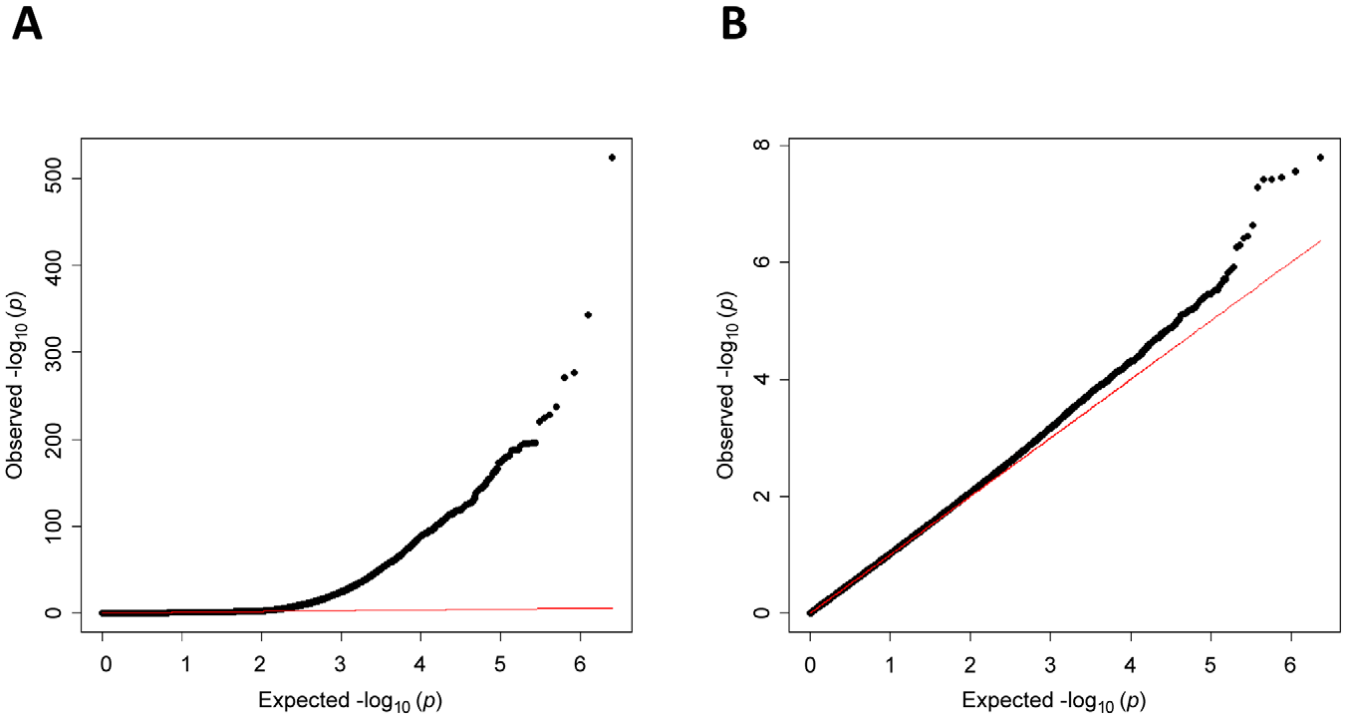
Quantile–quantile plot of *P*-values in meta-analysis for total white blood count. Panel A includes all markers, panel B excludes markers on chromosome 1. Horizontal axis indicates expected −log_10_ *P*-values. Vertical axis indicates observed −log_10_ *P*-values. The red line represents *y* = *x*. The marked deviation from expectations in panel A is due to markers on chromosome 1 near the *DARC* locus. The individual study ancestry-corrected inflation factors with (without) chromosome 1 markers were 1.06 (1.04) for ARIC, 1.02 (1.01) for CARDIA, 1.11 (1.08) for JHS, 1.03 (1.01) for Health ABC, 1.09 (1.05) for WHI, 1.04 (1.00) for GeneSTAR, and 1.01 (0.98) for HANDLS. The overall inflation factor prior to correction was 1.05 (0.98).

**Figure 2:**
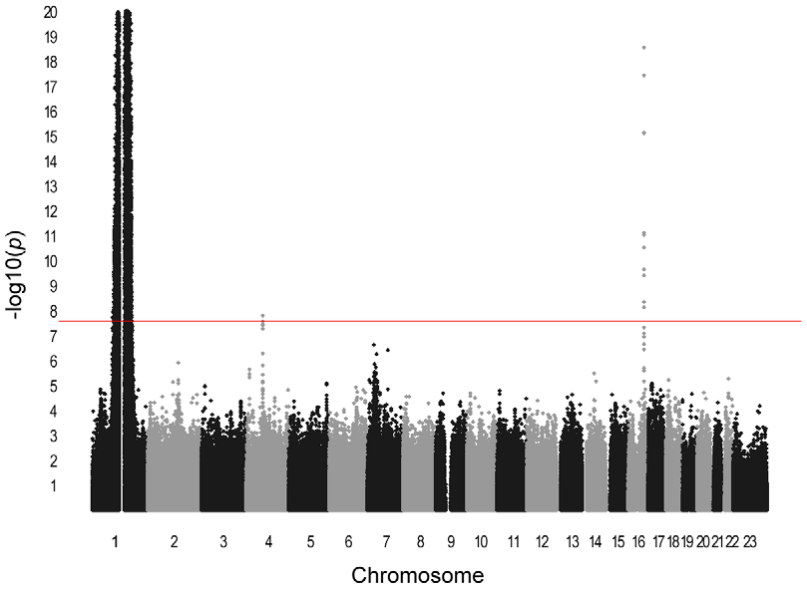
Manhattan plot of meta-analysis *P*-values in GWAS for total WBC count. Horizontal axis indicates chromosomal position. Vertical axis indicates −log10 *P*-values from inverse variance-weighted fixed effects meta-analysis. The red horizontal line indicates the genome-wide significance threshold of *P* = 2.5×10^−8^. Association signals are present at 1q23, 4q13, and 16q22. The *P*-values for the broad chromosome 1 signal are truncated at 10^−20^. This region spans nearly 90 Mb on both arms of chromosome 1 and results artifactually in two apparently distinct peaks because of the lack of genotyped or imputed SNPs around the centromere.

We performed GWA analysis of total WBC in an African-American discovery sample of 16,388 individuals from 7 population-based cohorts from the Continental Origins and Genetic Epidemiology Network (COGENT). The characteristics of each cohort are summarized in Table 1. Following stringent genotyping and imputation quality control procedures, a total of at least 2.4 million autosomal SNPs were available for analysis in each cohort (Table S1). Summary-level study results were combined by using inverse variance-weighted meta-analysis. The genomic-control corrected QQ plot for the combined African-African GWA analysis is shown in Figure 1. As summarized in Table 2, Table S2, and the Manhattan plot in Figure 2, three regions on chromosomes 1q23, 4q13, and 16q22 reached genome-wide significance at the threshold of *P*<2.5×10^−8^. These 3 loci are described in further detail below. Additional GWA analyses were performed on a subset of up to 7,477 COGENT African American participants with data available on WBC subtype counts (neutrophils, lymphocytes, monocytes, eosinophils and basophils) (Figures S1 and S2, Tables S3, S4, S5, S6, S7). Apart from the association of the chromosome 1q23 *DARC* locus with neutrophils and monocytes [6] (see below and Table 3), there were no new genome-wide significant associations (all *P*>2.5×10^−8^) for these phenotypes. African American cohort-specific results for index SNPs newly discovered or confirmed to be associated with WBC phenotypes are summarized in Figure S3 (total WBC), Figure S4 (neutrophil count), and Table S8.

**Table 1:** Characteristics of Continental Origins and Genetic Epidemiology Networks (COGENT) African-American discovery GWAS participants (n = 16,388).

Study	Atherosclerosis Risk in Communities (ARIC)	Coronary Artery Risk Development in Young Adults (CARDIA)	Johns Hopkins Genetic Study of Atherosclerosis Risk (GeneSTAR)	Healthy Aging in Neighborhoods of Diversity across the Life Span (HANDLS)	Health, Aging, and Body Composition (Health ABC)	Jackson Heart Study (JHS)	Women's Health Initiative (WHI)
Sample size	2664	943	934	862	898	1992	8095
Study design	Population-based, unrelated	Population-based, unrelated	Population-based, family	Population-based, unrelated	Population-based, unrelated	Population-based, unrelated	Population-based, unrelated
Age, years (SD)	53.4 (5.8)	24.4 (3.8)	45.2 (12.6)	48.2 (9.0)	73.4 (2.8)	50.0 (12.1)	61.6 (7.0)
% Female	63.2	58.7	61.6	56.0	58.8	61.2	100
WBC (SD)	5.67 (1.85)	5.92 (2.00)	6.00 (1.90)	6.20 (1.97)	5.60 (2.00)	5.68 (1.91)	6.49 (1.50)
Neutrophils (SD)	2.89 (1.45)	3.11 (1.57)	NA	3.485 (1.50)	5.20 (1.80)*	3.15 (1.51)	NA
Lymphocytes (SD)	2.17 (0.75)	2.23 (0.84)	2.24 (0.71)	2.08 (0.69)	1.80 (0.70)*	1.96 (0.66)	NA
Monocytes (SD)	0.344 (0.197)	0.314 (0.184)	0.308 (0.16)	0.386 (0.148)	0.400 (0.100)*	0.390 (0.145)	NA
Eosinophils (SD)	0.165 (0.165)	NA	NA	0.150 (0.099)	0.200 (0.100)*	0.139 (0.126)	NA
Basophils (SD)	0.037 (0.043)	0.046 (0.044)	NA	0.026 (0.014)	0.055 (0.040)*	0.033 (0.022)	NA

WBC and sub-type measures are reported in cells ×10^9^/liter [mean(SD)].

SD = standard deviation; NA = not available.

*In HABC, white blood cell sub-types were only available in a subset of participants (n = 207).

**Table 2:** Results of genome-wide significant SNPs for total white blood cell count.

Chromosome	Number SNPS with *P*<2.5×10^−8^	Top SNP in region	Position	Candidate gene	Minor/Major allele	Minor allele frequency	Effect size (Standard Error)	P-value
1q23	>10,000	rs2814778	157441307	*DARC*	T/C	0.21	+0.230 (0.005)	1.0×10^−524^
4q13	1	rs9131	75181913	*CXCL2*	T/C	0.23	−0.023 (0.004)	1.6×10^−8^
16q22	14	rs12149261	69555646	*HYDIN*	A/C	0.25	+0.083 (0.005)	3.5×10^−75^

For each locus, the lead SNP with the smallest *P*-values among the genotyped or imputed SNPs are indicated. Effect size represents the effect of a minor allele on natural log-transformed white blood cell count. Positions of the SNPs were derived from dbSNP build 136.

**Table 3:** Meta-analysis results of genome-wide significant SNPs for white blood cell count subtypes.

Cell type	Chromosome 1q23 *DARC* rs2814778 T allele			Chromosome 4q13 *CXCL2* rs9131 T allele		
	N	Effect size (Standard Error)	P-value	N	Effect size (Standard Error)	P-value
Neutrophils	5609	+0.305 (0.009)	1.0×10^−237^	7353	−0.038 (0.008)	1.5×10^−6^
Lymphocytes	5642	+0.020 (0.007)	3.8×10^−3^	7390	+0.010 (0.005)	0.06
Monocytes	5593	+0.048 (0.004)	6.0×10^−27^	7330	−0.004 (0.004)	0.23
Eosinophils	5411	+0.012 (0.003)	8.6×10^−5^	6402	−0.0005 (0.003)	0.85
Basophils	5104	+0.002 (0.0008)	3.5×10^−3^	6052	−0.0007 (0.0007)	0.27

Effect size represents the effect of a minor allele on natural log-transformed white blood cell count.

### Validation of *DARC* region on chromosome 1q23 as WBC–associated locus in African Americans

The GWA association signal on chromosome 1 is comprised of a broad peak encompassing 4,649 genotyped and imputed SNPs that exceeded the threshold of genome-wide significance. This region spans nearly 90 Mb on both arms of chromosome 1 (90,385,392–177,814,914 bp) and is approximately centered around the centromere. This results artifactually in two apparently distinct peaks in the Manhattan plot (Figure 2) because of the lack of genotyped or imputed SNPs around the centromere. Based on the 99% confidence interval of the distribution of test statistics, the strongest region of association is concentrated between position 155,127,086 and 160,217,075 on the short arm of chromosome 1 (*P* = 10^−154^ to 10^−524^). This region is centered around the *DARC* gene locus on 1q23.2. *DARC* contains rs2814778 (the Duffy null allele), previously identified as the likely causal chromosome 1q WBC-associated polymorphism in an admixture mapping study performed in the JHS and Health ABC cohorts, and confirmed in ARIC [4], [5]. As previously reported [4], [5], the *DARC* rs2814778 association with WBC is most consistent with a dominant rather than an additive model (*P* for dominance deviation <10^−40^). For example, in the largest cohort (WHI), the mean age- and global ancestry-adjusted WBC count was 4,823±1,004/µl in homozygotes for the African null allele, 6,307±1,006/µl in heterozygotes, and 6,563±1013/µl in homozygotes for the European wild-type allele.

Because the magnitude of the *DARC* rs2814778 polymorphism association might obscure any additional association signals present on chromosome 1, we repeated the GWAS analysis conditioning on the Duffy null rs2814778 polymorphism. All chromosome 1 SNPs which were significantly associated with WBC prior to rs2814778 adjustment became non-significant conditional on rs2814778 genotype (data not shown). When the association analysis was conducted separately for each white cell subtype, the *DARC* rs2814778 polymorphism was most strongly associated with the number of circulating neutrophils (P<10^−236^) (Table 3), but was also associated with the numbers of circulating monocytes (*P*<10^−26^), and to a lesser extent, lymphocytes, eosinophils, and basophils.

### *HYDIN* region association on chromosome 16q22 is most likely due to genotyping artifact

On chromosome 16q22, 13 SNPs spanning a ∼250 kb region (bp 69474507–69726247) that includes part of the large *HYDIN* gene locus were significantly associated with WBC. The lead SNP in the *HYDIN* region was rs12149261 (minor allele frequency or MAF 25%), an intronic polymorphism. The *HYDIN* association signal was confined to genotyped SNPs on the Affy6.0 array (ARIC, CARDIA, JHS, WHI). SNPs in this region were absent from the Illumina platform (Health ABC, GeneSTAR, HANDLS) and also absent from HapMap 2, thereby limiting imputation in the latter 3 cohorts.

Further examination of the sequence context in this region revealed that the *HYDIN* gene encompasses a large, recently duplicated segment of the genome, with a nearly identical 360-kb paralogous segment inserted on chromosome 1q21 [19], [20]. The chromosome 1q21 paralogue of the chromosome 16q22 segmental duplication is absent from build 36 of the NCBI human genome assembly. Nonetheless, 1q21 falls within the region encompassing the *DARC* association signal for WBC. Using genome-wide Affymetrix 6.0 genotype data from the ARIC African-American cohort, we determined the r-squared (pair-wise LD) between rs12149261 and every other typed SNP in the genome. There was reduced local LD within the chromosome 16 duplicated region, relative to the surrounding chromosome 16 SNP (Figure S5). Three SNPs had r-squared values of >0.20 with rs12149261: one located 20 kb away on chromosome 16 in the *HYDIN* gene (rs1774524; r2 = 0.27), and two located on chromosome 1 at ∼120 Mb near the *HYDIN* paralogue (rs12087334 and rs4659245; r2 = 0.25 and 0.22, respectively). Moreover, combined analysis of the 4 cohorts typed on the Affymetrix GWA platform showed that the chromosome 16q22 association signal at rs12149261 (*P* = 2.12×10^−18^) was completely abolished after conditioning on chromosome 1 *DARC* rs2814778 (*P* = 0.36). While defects in the *HYDIN* gene result in hydrocephalus [19], [20], this genomic region has not previously been associated with WBC. Together, these results demonstrate that the chromosome 16 *HYDIN* association finding is most likely a probe cross-hybridization artifact due to inter-chromosomal sequence similarity with the duplicated segment on chromosome 1q21 near the *DARC* region and that the polymorphisms associated with WBC in the studies using the Affymetrix arrays actually map to the chromosome 1 region.

### Discovery of a novel *CXCL2* association finding on chromosome 4q13 and replication in other ethnic populations

**Figure 3:**
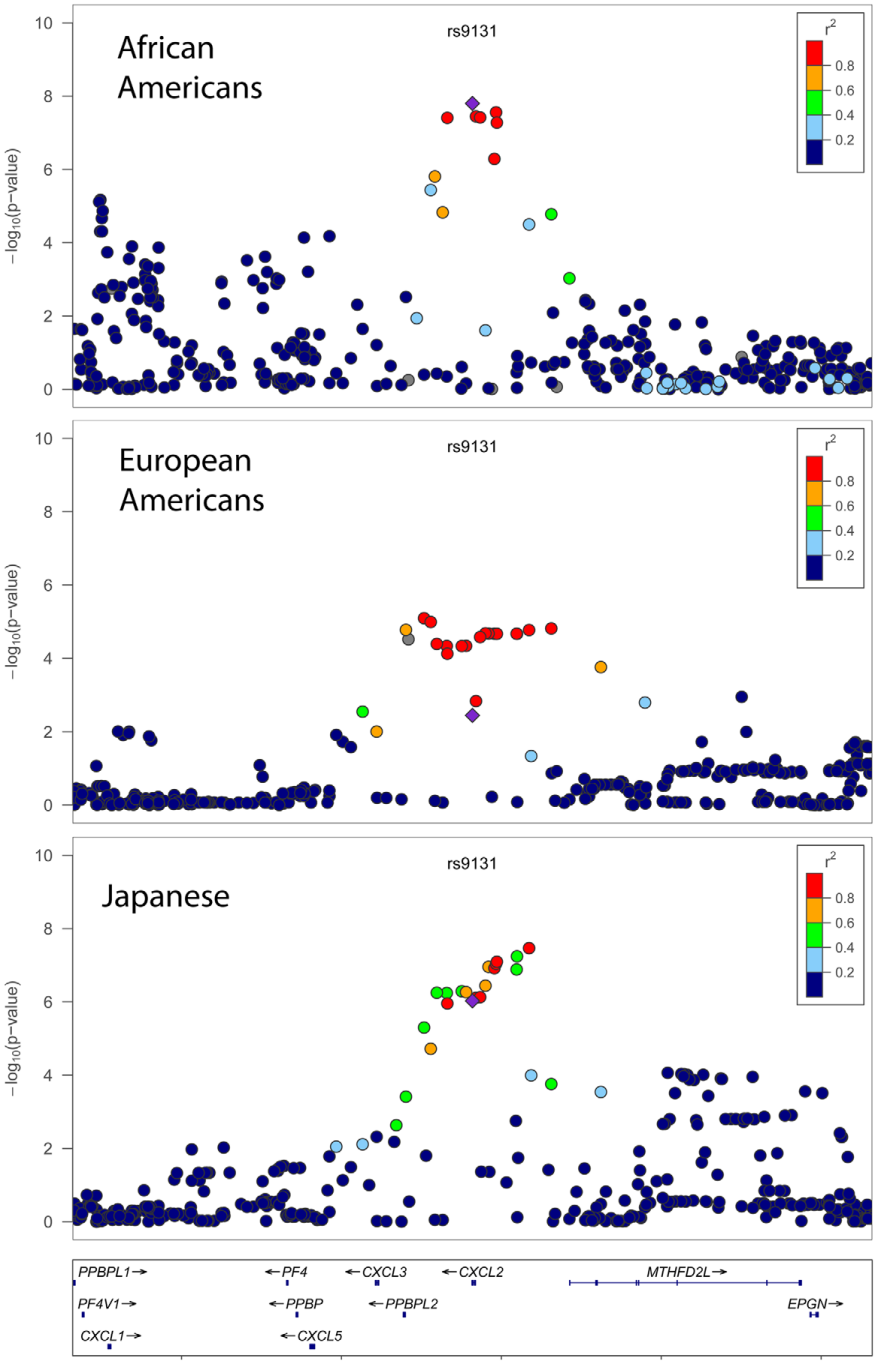
Population-specific −log P values for total WBC association at the chromosome 4q13 *CXCL2* locus. Shown is a 500 kb window of chromosome 4q13 centered at rs9131 (purple square). Plots were generated for African Americans (top panel), European Americans (middle panel), Japanese (bottom panel), using LocusZoom. The color of each SNP indicates the level of pairwise linkage disequilibrium (r-squared) relative to the index SNP rs9131. R-squared values were calculated from HapMap (release 22) YRI (top panel), CEU (middle panel), or CHB+JPT (bottom panel) populations. SNPs with missing LD information are shown in grey.

A novel SNP association on chromosome 4q13 was identified in our African-American WBC discovery GWAS. The lead SNP rs9131 is located in the 3′ UTR of the *CXCL2* gene, which encodes a macrophage-derived chemotactic cytokine for polymorphonuclear leukocytes. In African Americans, the minor T allele (MAF = 23%) was associated with lower WBC. Several additional SNPs in the chromosome 4 chemokine gene cluster had *P*-values ranging from 10^−5^ to 10^−7^, including rs2367291 located upstream of *CXCL1* (Figure 3A) Further adjustment for rs9131, however, abolished these associations (data not shown). Based on HapMap phase 2 and 1000 genomes data, rs9131 is in perfect LD with 7 other inter-genic SNPs in this region. Analysis of the subset of COGENT study participants with data available for number of circulating white cell subtypes indicated the rs9131 association was confined to neutrophils (Table 3).

To assess the role of the newly identified *CXCL2* association in other ethnic populations, we performed *in silico* replication using 3 samples: 3,551 Hispanic-American women from WHI-SHARe, 19,509 European-American participants from the CHARGE consortium, and 14,767 Japanese subjects from RIKEN. In Europeans, Hispanics, and Japanese, the T allele of rs9131 (frequency = 65%, 62%, and 46%, respectively) was associated with lower WBC (*P* = 0.004, 0.002, and 9.4×10^−7^, respectively), as was seen in African Americans (*P* = 2×10^−8^). The direction and magnitude of association was consistent across racial/ethnic groups: 0.009±0.003, 0.018±0.006, and 0.013±0.003 natural log units lower in Europeans, Hispanics, and Japanese, respectively, compared to 0.023±0.004 natural log units lower WBC count in the African-American discovery sample. Pooling the results across populations using a random effects meta-analysis gave a combined effect estimate (beta for lnWBC) of −0.015 (95%CI = −0.009 to −0.021) for rs9131. The *P* for Cochrane's Q test for heterogeneity was 0.04, with an *I^2^* of 64%. In contrast, there was no evidence that the chromosome 1 *DARC* region was associated with WBC count in either European or Japanese populations (data not shown).

**Figure 4:**
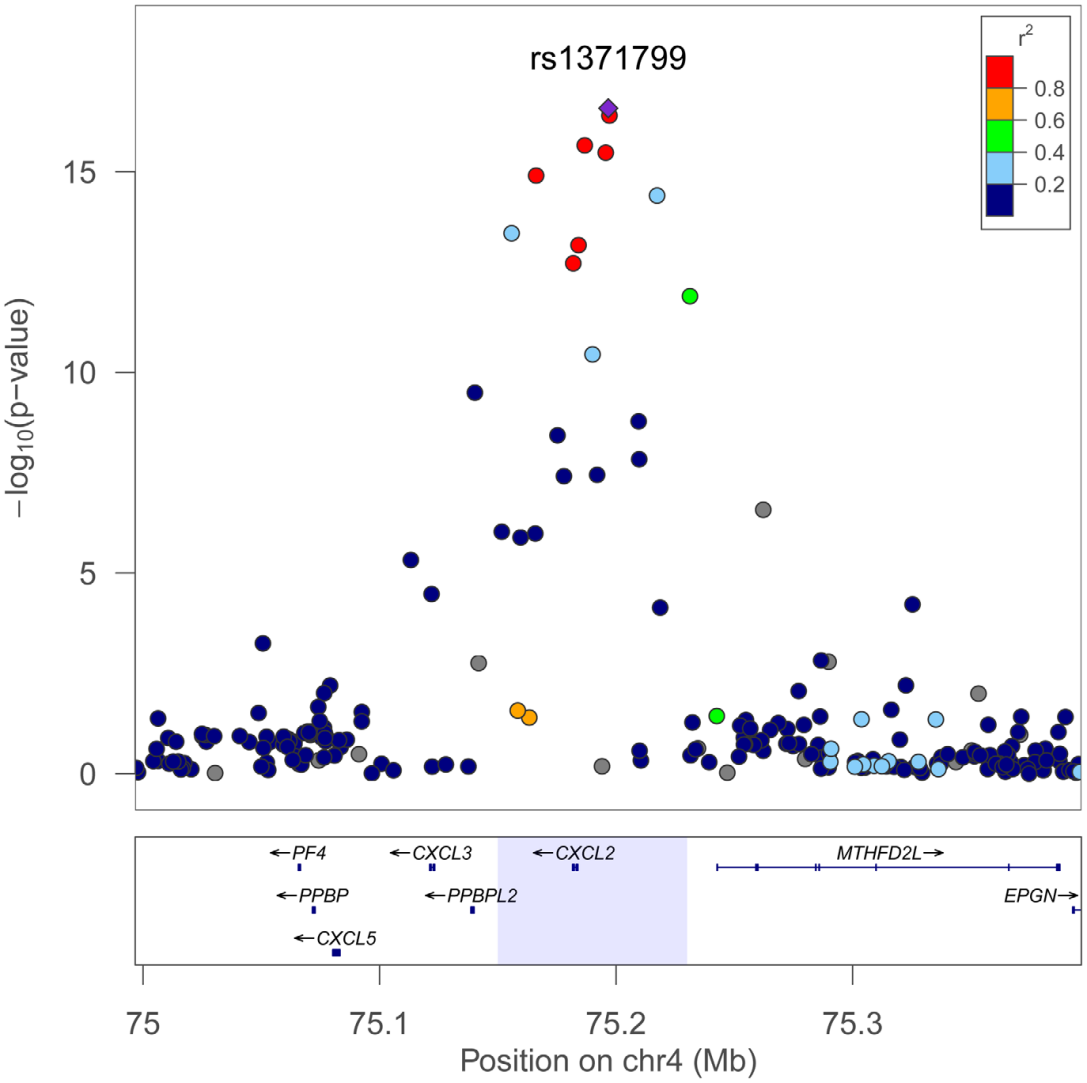
Trans-population meta-analysis results for total WBC count at the chromosome 4q13 *CXCL2* locus. The strongest association signal is localized to an LD bin of several SNPs within the *CXCL2* promoter and 5′ flanking region, including rs1371799 (purple triangle). Meta-analysis was performed using Fisher's method to combine P-values across African, European, and Japanese populations. The 99% confidence interval for the cross-population association signal mapped to a 75 kb region shaded in light blue (lower panel). Plot was generated using LocusZoom. Linkage disequilibrium is shown for the African population.

Regional plots comparing the SNP association and linkage disequilibrium patterns across *CXCL2* on chromosome 4 in African Americans, Europeans, and Japanese 4 are shown in Figure 3. In Europeans and Japanese, several additional SNPs in the *CXCL2* region of chromosome 4 had stronger association WBC signals than rs9131. Specifically, rs16850408, which is located in an inter-genic region between *CXCL2* and the pro-platelet basic protein-like 2 gene (*PPBPL2*), was most strongly associated with WBC (*P* = 8.04×10^−6^) in Europeans. The r-squared between rs16850408 and rs9131 is 0.76 in European and 0.3 in African HapMap samples. In Japanese, rs7686861 located in the intergenic region between *CXCL2* and *MTHFD2L* (methylenetetrahydrofolate dehydrogenase 2-like) was the lead SNP (*P* = 3.4×10^−8^). The r-squared between rs7686861 and rs9131 is 0.21 in Asian and 0.23 in African HapMap samples. To further narrow the locus of WBC count association, we performed a sample size-weighted meta-analysis of the *CXCL2* region across all 3 ethnic groups. The cross-population association signal mapped to a 75 kb region (positions 75,155,842–75,231,250), which contains *CXCL2* and no other genes in the chromosome 4q13 region. The top SNPs included rs1371799 (*P* = 1.7×10^−17^) as well as several others located within the *CXCL2* promoter and 5′ flanking region (Figure 4).

### Assessment of other previously discovered WBC–associated loci in African-Americans

Several GWAS loci have been published from European or Japanese cohorts, including those associated with WBC (*GSDMA-ORMDL3-PSMD3-CSF3*, *HSB1L-MYB*, *CDSN-PSORS1C1*, *CDK6*, and *RAP1B*), neutrophil count (*PSMD3-CSF3*, *PLCB4*), and eosinophil count (*IL1RL1*, *IKZF2*, *GATA2*, *IL5*, *SH2B3*) [12]–[15]. Table 4 shows the association results of these same loci in our African-American sample, for the originally reported index SNP. Extending the association analyses to SNPs in LD with the index SNP (*r^2^*≥0.5 in HapMap CEU or CHB+JPT) did not reveal any additional associations (data not shown). For the chromosome 17 *PSMD3–CSF3* region, the T allele of rs4065321 reported to be associated with lower WBC in Japanese was similarly associated with lower total WBC in African-Americans (*P* = 1×10^−4^). Most of the African-American WBC-associated SNPs in this region were intronic to *PSMD3*, while one SNP (rs7224260) is located in the 3′ flanking region of *CSF3*. The T allele of *CDK6* rs445 was associated with lower total WBC (Table 4), and also with lower neutrophil count in 7,392 African Americans (beta −0.0249±0.0049; p = 1.7×10^−7^). The remaining European and Japanese WBC-association genomic regions listed in Table 4 showed little evidence of replication in African Americans.

**Table 4:** Assessment in African-Americans of loci previously associated with leukocyte traits in Caucasians and/or Japanese.

					European or Japanese Discovery Sample			COGENT African Americans		
Trait	Discovery Population [Ref.]	Chromosome (Position)	Locus	Lead SNP	Sample size	effect allele (frequency)	BETA ± SE (P-value)	Sample size	effect allele (frequency)	BETA ± SE (P-value)
WBC	European [12]	17 (35,364,215)	*GSDMA-ORMDL3-CSF3*	rs17609240	13,943	T (0.26)	−0.019±0.003 (9×10^−9^)	14628	T (0.23)	−0.004±0.005 (0.35)
**WBC**	**Japanese ** [**13**]	**17 (35,397,074)**	***GSDMA-ORMDL3-CSF3***	**rs4065321**	**14,677**	**T (0.32)**	**−0.094±0.012 (3×10^−14^)**	**16388**	**T (0.63)**	**−0.014±0.004 (1.3×10^−4^)**
WBC	Japanese [13]	6 (135,468,266)	*HSB1L-MYB*	rs4895441	14,677	G (0.37)	−0.073±0.012 (2×10^−9^)	16351	G (0.10)	−0.0002±0.006 (0.98)
WBC	Japanese [13]	6 (31,193,749)	*CDSN-PSORS1C1*	rs3094212	14,677	C (0.35)	0.070±0.012 (7×10^−9^)	16295	C (0.33)	−0.004±0.004 (0.32)
**WBC**	**Japanese ** [**13**]	**7 (92,246,306)**	***CDK6***	**rs445** ¶	**14,677**	**T (0.32)**	**−0.070±0.013 (2×10^−8^)**	**16388**	**T (0.19)**	**−0.025±0.005 (3.8×10^−7^)**
WBC	Japanese [13]	12 (67,247,851)	*RAP1B*	rs12313946	14,677	C (0.47)	0.065±0.012 (3×10^−8^)	16388	C (0.77)	0.007±0.004 (0.062)
Neutrophils	Japanese [14]	17 (35,410,238)	*GSDMA-ORMDL3-CSF3*	rs4794822	7,665	C (0.48)	−0.101±0.016 (6×10^−10^)	7401	C (0.66)	−0.048±0.073 (0.50)
Neutrophils	Japanese [14]	20 (9,313,303)	*PLCB4*	rs2072910	7,665	C (0.30)	−0.111±0.018 (3×10^−10^)	7401	C (0.57)	0.003±0.007 (0.95)
Eosinophils	European [15]	2 (102,324,148)	*IL1RL1*	rs1420101	21,510	A (0.41)	6.4±0.87* (5.3×10^−14^)	6437	A (0.32)	0.004±0.002 (0.052)
Eosinophils	European [15]	2 (213,532,290)	*IKZF2*	rs12619285	21,510	G (0.74)	6.3±1.02* (5.4×10^−10^)	6381	G (0.33)	−0.005±0.002 (0.032)
Eosinophils	European [15]	3 (129,743,240)	*GATA2*	rs4857855	21,510	T (0.82)	9.4±1.12* (8.6×10^−17^)	6437	T (0.15)	−0.005±0.003 (0.091)
Eosinophils	European [15]	5 (131,890,876)	*IL5*	rs4143832	21,510	C (0.16)	7.1±1.07* (1.2×10^−10^)	6328	C (0.63)	0.0001±0.002 (0.95)
Eosinophils	European [15]	12 (110,368,991)	*SH2B3*	rs3184504	21,510	T (0.38)	7.6±0.87* (6.5×10^−19^)	6437	T (0.07)	0.007±0.004 (0.070)

Genomic positions and annotations are given using NCBI build 36.1. Effect allele is always on the forward strand. Effect size (BETA) and standard error (SE) are given a natural log-transformed WBC. The replicated loci are highlighted in bold font.

*In reference [15], effect sizes were reported in percentages of standard deviation units.

**¶:** *CDK6* rs445 was also associated with −0.0249±0.0049 lower neutrophil count in n = 7392 African Americans (*P* = 1.7×10^−7^).

### Effect of locus-specific ancestry on newly and previously reported WBC–associated SNPs

In recently admixed populations, it is possible that confounding of a SNP association may occur as a result of local as well as global differences in genetic ancestry between study participants [21]. Therefore, we repeated the association analyses for any newly reported African American or previously reported European and Japanese genome-wide significant WBC-associated loci, additionally adjusting for estimated local ancestry in our COGENT African American study participants. We performed these locus-specific ancestry conditional analyses in a subset of 13,694 participants from each of the 4 cohorts genotyped on Affymetrix 6.0 (WHI, ARIC, CARDIA, and JHS). After meta-analyzing the African American cohort-specific results, there was essentially no difference between the local ancestry adjusted versus global ancestry-adjusted associations at any of the WBC-associated loci (Table S9). However, when we performed an additional association analysis for each lead SNP stratifying on the estimated local number of European versus African chromosomes, the *CDK6* rs445 and *PSMD3-CSF3* rs4065321 WBC associations were stronger on a local European ancestral background than on an African background (Table S9). Notably, the *CDK6* and *PSMD3-CSF3* loci are also the only two previously reported WBC associations that we were able to replicate in our African American sample. For European and Japanese WBC-associated loci that didn't replicate in our African American sample, there was no evidence of any differential association according to local ancestral background or proportion of European ancestry in the AA sample (data not shown).

### Heritability of WBC phenotypes in African Americans and proportion of variance explained

 Polygenic heritability was estimated for unadjusted and age- and sex-adjusted total WBC, neutrophil, lymphocyte, and monocyte count using 236 African-American pedigrees from the GeneSTAR study (Table S10). All WBC phenotypes showed significant heritability (*P*<0.001). The heritability estimates ranged from 48–49% for total WBC and neutrophil count to ∼29% for monocyte count. The proportion of total variance explained by *DARC* rs2814778+*CXCL2* rs9131+*CDK6* rs445+*PSMD3-CSF3* rs4065321 in the COGENT African American cohorts ranged from 16% to 24% for WBC, 20% to 25% for neutrophils, and 2% to 7% for monocytes.

Since multiple, independent variants at the same locus may account for some of the “missing heritability” of complex traits [22], we repeated the association tests for all genotyped SNPs within 500 kb of the *DARC*, *CXCL2*, *CDK6*, and *PSMD3-CSF3* gene regions for WBC association, conditioning on the lead SNP in each region. None of the 4 loci contained additional SNPs associated with WBC at *P*<2.5×10^−5^ (a Bonferroni-corrected significance threshold calculated from the 2,000 SNPs tested in these 4 regions).

## Discussion

Recently the African null allele of rs2814778 at the Duffy Antigen Receptor for Chemokines locus on chromosome 1 was found to be associated with lower total leukocyte and neutrophil counts in African Americans [4]–[6]. By screening 16,388 African-American participants, we have confirmed the strong *DARC* association. We also identified a second chemokine-related gene region associated with lower WBC, with the lead SNP rs9131 located in the *CXCL2* gene. Independent evidence of the novel *CXCL2* association was present in other ethnic populations, including ∼3,500 Hispanic Americans, ∼15,000 Japanese, and ∼20,000 European Americans. Two additional WBC loci recently identified through GWAS of European or Japanese populations (*CDK6* gene region on chromosome 7 and *PSMD3-CSF3* region on chromosome 17 [12]–[14] were associated with WBC traits in African Americans. We also demonstrate that large inter-chromosomal duplications can result in false positive associations in GWAS as was shown for *HYDIN*.

Our estimate of heritability for total WBC and neutrophil count in African Americans was close to 50%, which is similar to that reported in European populations [16]–[18]. While our GWAS has identified a few, select loci to be associated with WBC count in African Americans, the proportion of variation explained for WBC and neutrophil count was still less than 25%, and considerably lower for the remaining WBC subtypes. Therefore it seems likely that in addition to the *DARC* and *CXCL2* loci, other yet-to-be identified loci exist. Alternatively, genetic factors may account for a lower percentage of the variance in WBC count than suggested by heritability estimates and perhaps environmental factors should be more broadly considered. Other factors may have limited our ability to identify genetic mechanisms underlying these traits, including phenotype measurement error and reduced sample size and power for the WBC subtype GWA analyses. Multiple rare genetic variants or gene-gene and gene-environment interaction may also account for some of the inter-individual variation of these hematologic traits.

Myelopoiesis is regulated by a number of cytokines, chemokines, growth factors, and their receptors. The cytokine granulocyte colony-stimulating factor (G-CSF), encoded by the *CSF3* gene on chromosome 17, is critically involved in granulopoiesis by stimulating proliferation, differentiation, and survival of neutrophil precursors [23] and by regulating the rate of release of neutrophils from the bone marrow under non-inflammatory conditions [24]. During infection or inflammation, neutrophil, monocyte and eosinophil mobilization from the bone marrow can occur through the systemic and/or local action of several chemokines, which stimulate chemotaxis across the bone marrow sinusoidal endothelium. G-CSF stimulates neutrophil mobilization and release by down-regulating signaling of stromal-derived factor 1 (CXCL12) through its receptor CXCR4, which serves as a bone marrow retention signal for mature neutrophils [23], [25]. In contrast, the chemokines CXCL1 and CXCL2, by binding to CXCR2, promote rapid release of neutrophils from the bone marrow, thereby elevating blood neutrophil counts during infection or during G-CSF-induced neutrophil mobilization [25]–[27].

DARC is selectively expressed on red blood cells and venular endothelial cells and binds several pro-inflammatory chemokines of both the CXC and CC subfamilies. Endothelial DARC facilitates leukocyte recruitment and trans-endothelial migration, thereby contributing to inflammatory disease pathogenesis and severity in animal models [28]–[30]. Erythrocyte DARC has been proposed to act as a chemokine scavenger, sink or reservoir, maintaining basal plasma chemokine concentrations, though the biological relevance of this sink function remains unclear [6]–[9]. The African Duffy null variant disrupts a *DARC* promoter binding site for the transcription factor GATA-1, and results in complete absence of DARC from erythrocytes without affecting endothelial DARC expression [31]. Duffy-negative individuals are protected from *P. vivax* malaria [32], [33] and have been reported to have a survival advantage in leukopenic HIV-infected persons of African descent [34]. Interestingly, during systemic inflammation neutrophils from DARC-deficient mice exhibit impaired chemotaxis toward CXCL2 that appears to result from altered plasma chemokine levels and down-regulation of neutrophil CXCR2 expression [29]. It is conceivable that a homeostatic role of DARC in CXCL1/CXCL2- CXCR2 chemokine ligand-receptor interactions during inflammation may also extend to the setting of neutrophil release from the bone marrow under both basal and inflammatory conditions.

Nucleotide diversity can vary substantially across populations due to different evolutionary histories and migration patterns. Generally, nucleotide diversity is greatest and linkage disequilibrium lowest among African populations. By leveraging the extent of variation in LD patterns between populations, localization of causal variants can be improved by analyzing multiple ethnic groups [35]–[38]. By combining WBC count association results from the *CXCL2* region across African Americans, European Americans, and Japanese, we were able to narrow the association signal to the *CXCL2* promoter and 5′ flanking region.

The multi-gene region on chromosome 17q21.1 has now been associated with WBC or neutrophil count in Europeans [12], Japanese [13], [14], and African Americans. The index SNPs originally reported (rs17609240, rs4065321, rs4794822, rs2305481) for these traits are in strong to moderate LD in Europeans and Japanese (*r* ^2^ = 0.5 to 1.0), spanning an LD block that includes several genes (*GSDMA*, *ORMDL3*, *PSMD3*, *CSF3*, *MED24*, *SNORD124*, and *THRA*). The lower extent of LD in African-Americans suggests finer localization of the rs4065321 WBC-associated signal to the region containing *PSMD3* and *CSF3*. Other variants in this region have been associated with childhood-onset asthma [39]. *CSF3*, which encodes G-CSF, constitutes the most likely biologic candidate in this region responsible for phenotypic variation in WBC. However, the functional SNPs responsible for variation in WBC phenotypes remain to be identified. Expression (eQTL) analysis demonstrated that the SNP associated with neutrophil count by Okada et al was associated with *PSMD3* expression, rather than *CSF3* expression [14]. *PSMD3* encodes one of the non-ATPase subunits of the 19S regulator of 26S proteasome, which is involved in regulation of the cell cycle through the ubiquitin–proteasome pathway.

The current analysis also replicated the association between WBC count and a region on chromosome 7 containing the gene for CDK6, or cyclin-dependent kinase 6, another regulator of cell cycle progression known to be expressed in proliferating hematopoietic progenitor cells [40]. Through its interaction with the transcription factor Runx1, CDK6 inhibits terminal granulocytic differentiation [41]. For the chromosome 7 WBC locus, rs445 is located within the first intron of *CDK6*, and represents the lead SNP in both Japanese [13] and our African American sample. There is no other variant in strong LD (r-squared>0.8) with rs445 in any HapMap or 1000 Genomes population. Therefore it is possible that *CDK6* rs445 may represent the actual causal variant. Other polymorphisms within the *CDK6* gene have been associated with susceptibility to rheumatoid arthritis [42] and height [43].

Benign neutropenia is defined as an absolute neutrophil count (ANC) of less than 1.5×10^9^ cells/L on repeated occasions [2], [44]. It occurs in up to 40% of individuals of African descent [2] and is present in ∼5% of adult African Americans compared to <1% of European Americans [3]. The benign neutropenia of African Americans is characterized by normal myeloid maturation, but slightly reduced numbers of bone marrow myeloid progenitors [45], [46] and reduced numbers of mature neutrophils that can be released from bone marrow stores [47]. Despite having slightly lower steady-state bone marrow CD34^+^ hematopoietic progenitor cells, African Americans paradoxically appear to have enhanced peripheral blood stem-cell mobilization in response to administration of G-CSF compared to whites [44], [48]. The genetic determinants of these features of G-CSF-induced stem cell mobilization remain to be determined.

In summary, polymorphisms within *DARC* on chromosome 1 and *CXCL2* on chromosome 4, and near *CDK6* on chromosome 7 and *CSF3* on chromosome 17, are associated with WBC in African Americans. These findings contribute to our understanding of genetic factors underlying variation in WBC within and between populations and highlight the importance of common genetic variants in genomic regions encoding chemokine ligands and receptors to regulation of myelopoiesis and circulating leukocyte counts in human populations. Further localization and characterization of the functional variants responsible for these WBC and neutrophil associations could help to inform clinical approaches to cancer-associated neutropenia or hematopoietic stem cell mobilization.

## Methods

### Subjects

The subjects participating in the GWAS consisted of a total of 16,388 self-identified African-American individuals from 7 population-based cohorts (ARIC, CARDIA, JHS, WHI, HANDLS, Health ABC, and GeneSTAR) that belong to the Continental Origins and Genetic Epidemiology Network (COGENT). Detailed descriptions of each participating COGENT cohort, their quality control practices and study-level analyses are provided in the Text S1. Clinical information of the subjects was collected by self-report and clinical examination. All participants provided written informed consent as approved by local Human Subjects Committees. We excluded study participants on the basis of pregnancy, cancer, or AIDS diagnosis at the time of blood count measurement.

### WBC phenotype data

Certified staff obtained fasting blood samples at the baseline clinic visit. Samples for complete blood count (CBC) analysis were obtained by venipuncture and collected into tubes containing ethylenediaminetetraacetic acid (EDTA). Total circulating WBC count and cell subtype counts were performed at local clinical laboratories using automated hematology cell counters and standardized quality assurance procedures [4], [6], [49]–[51]. Total WBC count was reported in millions of cells per ml, and was recorded in all 16,388 study participants. Information on WBC subtype was available only in a subset of 7,477 (45.6%) participants from ARIC, CARDIA, JHS, HANDLS, GeneSTAR, and Health ABC. WBC differentials were performed by clinically certified hematology laboratories. The absolute numbers of each type of WBC were calculated by multiplying the proportion of the WBC count comprised by each cell type by the total WBC measure. To evaluate normality of the phenotypes for subsequent regression analyses, we performed Box-Cox likelihood ratio tests on raw WBC phenotypes. On this basis, all WBC traits were natural log transformed to normalize the distributions of the phenotypic data.

### Genotype data and quality control

Genome-wide genotyping was performed within each COGENT cohort using methods described under Text S1. DNA samples with a genome-wide genotyping success rate <90%, duplicate discordance or sex mismatch, genetic ancestry outliers (as determined by cluster analysis performed using principal component analysis or multi-dimensional scaling), SNPs with genotyping success rate <95%, monomorphic SNPs, SNPs with minor allele frequency (MAF) <1%, and SNPs that map to several genomic locations were removed from the analyses. Significantly associated SNPs were examined for strong deviations from Hardy–Weinberg equilibrium and/or raw genotype data examined for abnormal clustering. Participants and SNPs passing basic quality control were imputed to >2.2 million SNPs based on HapMap2 haplotype data using a 1∶1 mixture of Europeans (CEU) and Africans (YRI) as the reference panel. Details of the genotype imputation procedure are described further under Text S1. Prior to discovery meta-analyses, SNPs were excluded if imputation quality metrics (equivalent to the squared correlation between proximal imputed and genotyped SNPs) were less than 0.50.

### Data analysis

For all cohorts, genome-wide association (GWA) analysis for quantitative WBC traits was performed using linear regression adjusted for covariates, implemented in either PLINK v1.07 [52] or MACH2QTL v1.08. Allelic dosage at each SNP was used as the independent variable, adjusted for primary covariates of age, age-squared, sex, and clinic site (if applicable). To adjust for population stratification and global admixture, the principal components were also incorporated as covariates in the regression models (see Text S1). For GeneSTAR, family structure was accounted for in the association tests using linear mixed effects (LME) models implemented in R [53]. Although the JHS has a small number of related individuals, extensive analyses showed that results were concordant using linear regression or LME, after genomic control. Therefore, results are presented for JHS using linear regression. For imputed genotypes, we used dosage information (*i.e.* a value between 0.0–2.0 calculated using the probability of each of the three possible genotypes) in the regression model implemented in PLINK and MACH2QTL (for cohorts with unrelated individuals) or the Maximum Likelihood Estimation (MLE) routines (for GeneSTAR).

For each WBC phenotype, meta-analyses were conducted using inverse-variance weighted fixed-effects models to combine beta coefficients and standard errors from study level regression results for each SNP to derive a combined p-value and effect estimates. Study level results were corrected for genomic inflation factors (λ) by incorporating study specific λ estimates into the scaling of the standard errors (SE) of the regression coefficients by multiplying the SE by the squate-root of the genomic inflation factor. The inflation factors for all completed analyses are presented in Table S1. Meta-analyses were implemented in the software METAL [54] and were performed independently by another analyst to confirm results. Between-study heterogeneity of results was assessed by using Cochran's *Q* statistic and the *I* ^2^ inconsistency metric. For each genome-wide significant or replicated locus, cohort specific-results and overall WBC effect estimates and confidence intervals are summarized using forest plots (Figure S3 and S4). The mean and standard deviation WBC count for each genotype class is provided in Table S8.

To maintain an overall type 1 error rate of 5%, a threshold of α = 2.5×10^−8^ was used to declare genome-wide statistical significance. This threshold has been suggested for African ancestry populations based on estimates of ∼2 million independent common variant tests in African genomes [55].

Given the nonlinear nature of the original phenotype, we performed a sensitivity analysis of whether our results are robust to the assumption of an additive genetic model. We repeated the GWA analysis for the WHI, ARIC, CARDIA, JHS cohorts, the four largest African American cohorts (n = 13,694) using a 2 degree of freedom genotypic model as well as a dominance deviation test, and meta-analyzed the results using METAL.

To assess in the COGENT African-Americans WBC trait-associated loci previously reported in Europeans or Japanese, we evaluated the African-American meta-analysis results for each index SNP in the regions reported, including consistency of direction of effect, and assessed statistical significance by a simple Bonferroni adjustment based on the total number of SNPs assessed using a 2-sided hypothesis test. In addition, we performed a more exploratory assessment of all SNPs within a 500 kb window that were correlated in African Americans with the European or Japanese index SNP in HapMap CEU or CHB+JPT (*r^2^*≥0.5). We adjusted these exploratory regional analyses for multiple testing based on the effective number of SNPs, taking into account pairwise linkage disequilibrium patterns.

To further assess the potential existence of multiple, independent variants influencing a trait at the same locus (allelic heterogeneity), regression analyses were repeated, conditional on the most strongly associated (index) SNP in that region. Each study repeated the primary GWA analysis, additionally adjusting for the lead SNP in each region under the appropriate regression models. The cohort-specific results were then meta-analyzed in the same way as for the primary GWA study using METAL.

### Replication and fine-mapping of new WBC association signals

Replication of novel association findings was performed using GWA data in 3 other ethnic populations: 3,551 Hispanic American women from WHI, 14,767 Japanese from RIKEN, and 19,509 European Americans from CHARGE. Further details of each study population are provided under Text S1. Both genotyped and imputed SNP data were available in the European and Japanese samples, while only genotyped SNP data were available in the Hispanic Americans. To further localize the causal variant responsible for the *CXCL2*-WBC association, we extended the association analysis to include all genotyped and imputed SNPs within a 500 kb region centered at rs9131, the SNP most strongly associated with WBC count in African Americans. We then performed a trans-population meta-analysis of each SNP in this region by combining test statistics from the African American (COGENT), European (CHARGE), and Japanese (RIKEN) association analyses using Fisher's method [56], which may have some advantages over the standard meta-analytic approach in this setting [37]. Nonetheless, we also performed a standard inverse variance-weighted meta-analysis using either fixed or random effects [57], and obtained results similar to Fisher's method.

### Local ancestry analyses

For between-study GWA platform consistency, we estimated locus-specific ancestry using Affymetrix 6.0 genotyped SNP data from the 4 largest African-American cohorts (WHI, ARIC, JHS, CARDIA), which constitute ∼85% of our total COGENT African American sample. For each African American, locus-specific ancestry (probabilities of whether an individual has 0, 1, or 2 alleles of African ancestry at each locus) was estimated using a Hidden Markov Model and local haplotype structure to detect transitions in ancestry along the genome [58], [59]. Phased haplotype data from the HapMap CEU and YRI individuals were used as reference panels. To assess the impact of local ancestry on any genome-wide SNP associations, each of the 4 cohorts repeated each SNP genotype-WBC phenotype linear regression model, adjusting for local ancestry proportion as a covariate. In addition, we stratified the SNP genotype-WBC phenotype association test on the number of estimated local European chromosomes (≥1 versus <1) to compare whether variants in genome-wide significant regions have the same versus different effect on African and European ancestral population backgrounds. The cohort-specific results of these analyses were combined using METAL.

### Heritability and proportion of variance explained

In the GeneSTAR family study, variance components models in the ASSOC subroutine of S.A.G.E. [60] were used to derive maximum likelihood estimates of polygenic (narrow-sense) heritability (*σ* ^2^_g_) using natural-log transformed unadjusted or covariate-adjusted phenotype data. The statistical significance of the heritability estimate was obtained using a likelihood ratio test. In each of the 7 COGENT African American cohorts, the fraction of variance explained was estimated using the formula: 2*pq*×*β* ^2^, where *p* is the frequency of the effect allele of the SNP, *q* = 1−*p*, and *β* is the additive effect in each population estimated by standardizing WBC to have standard deviation 1.

## Supporting Information

Figure S1 Quantile-quantile (QQ) plots of the meta-analyses for basophil, eosinophil, lymphocyte, monocyte, and neutrophil traits in African Americans.
(TIF)

Figure S2 Manhattan of plots for neutrophil, lymphocyte, monocyte, eosinophil, and basophil counts.
(DOC)

Figure S3 Forest plots of cohort-level and summary-level genotype risk estimates and confidence intervals for total WBC.
(TIF)

Figure S4 Forest plots of cohort-level and summary-level genotype risk estimates and confidence intervals for neutrophil count.
(TIF)

Figure S5 Linkage disequilibrium (LD) plots in the region of the chromosome 16 *HYDIN* locus.
(DOC)

Table S1 Summary of SNP information and genomic inflation factors for all association analyses performed in this study.
(DOC)

Table S2 Results of top SNPs (*P*<1×10^−5^) for total white blood cell count.
(XLS)

Table S3 Results of top SNPs (*P*<1×10^−5^) for neutrophils.
(XLS)

Table S4 Results of top SNPs (*P*<1×10^−5^) for monocytes.
(XLS)

Table S5 Results of top SNPs (*P*<1×10^−5^) for eosinophils.
(XLS)

Table S6 Results of top SNPs (*P*<1×10^−5^) for basophils.
(XLS)

Table S7 Results of top SNPs (*P*<1×10^−5^) for lymphocytes.
(XLS)

Table S8 Cohort-level mean and standard deviation WBC count for each genotype class at WBC–associated loci.
(XLSX)

Table S9 Locus-specific ancestry-adjusted analyses of WBC–associated loci.
(DOC)

Table S10 Heritability estimates of WBC phenotypes.
(DOC)

Table S11 Characteristics and distributions of traits in the study populations by the CHARGE Consortium.
(DOC)

Text S1 Supplemental Materials and Methods.
(DOC)
