# Supplementary figures and images for: Genome-Wide Association Study of White Blood Cell Count in 16,388 African Americans: the Continental Origins and Genetic Epidemiology Network (COGENT)
Source: PLoS Genet. 2011 Jun 30;7(6):e1002108. doi: 10.1371/journal.pgen.1002108 (PMC3128101; doi:10.1371/journal.pgen.1002108)

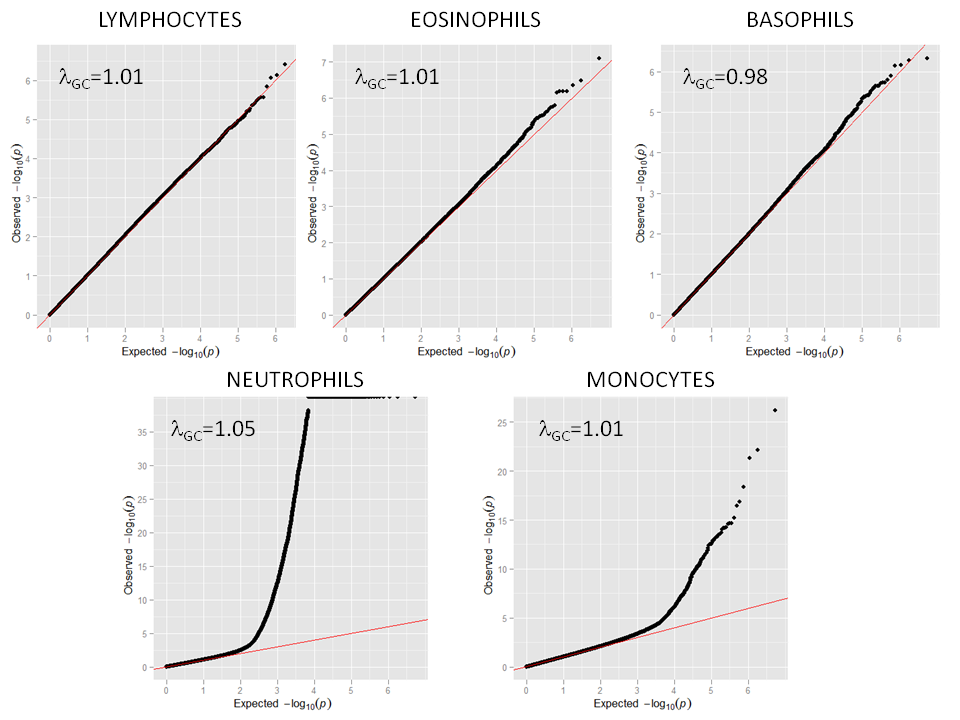

Supplement: Figure S1 — Quantile-quantile (QQ) plots of the meta-analyses for basophil, eosinophil, lymphocyte, monocyte, and neutrophil traits in African Americans. (TIF) [file pgen.1002108.s001.tif]

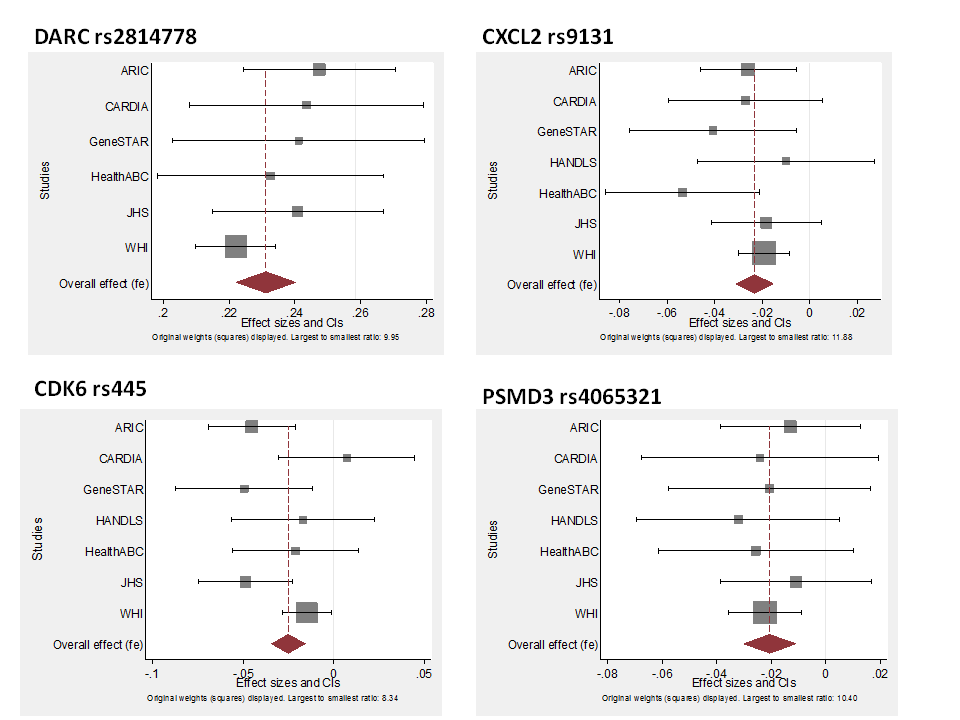

Supplement: Figure S3 — Forest plots of cohort-level and summary-level genotype risk estimates and confidence intervals for total WBC. (TIF) [file pgen.1002108.s003.tif]

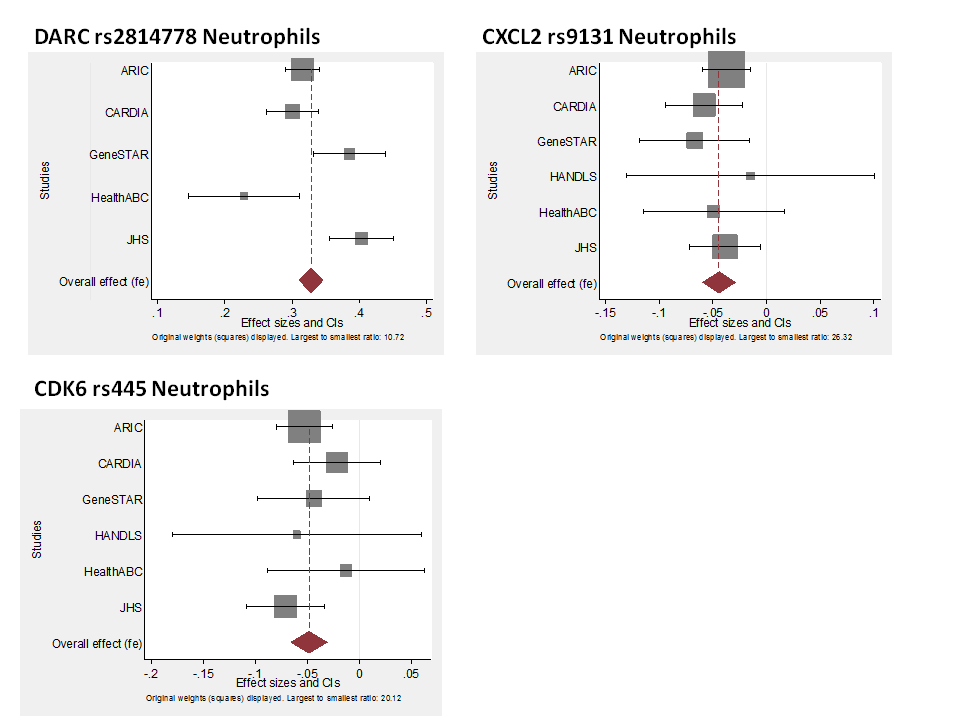

Supplement: Figure S4 — Forest plots of cohort-level and summary-level genotype risk estimates and confidence intervals for neutrophil count. (TIF) [file pgen.1002108.s004.tif]
