# Supplementary material for: Genome-Wide Association Study of White Blood Cell Count in 16,388 African Americans: the Continental Origins and Genetic Epidemiology Network (COGENT)
Source: PLoS Genet. 2011 Jun 30;7(6):e1002108. doi: 10.1371/journal.pgen.1002108 (PMC3128101; doi:10.1371/journal.pgen.1002108)

**Supplementary Figure 2.** Manhattan of plots for neutrophil, lymphocyte, monocyte, eosinophil, and basophil counts


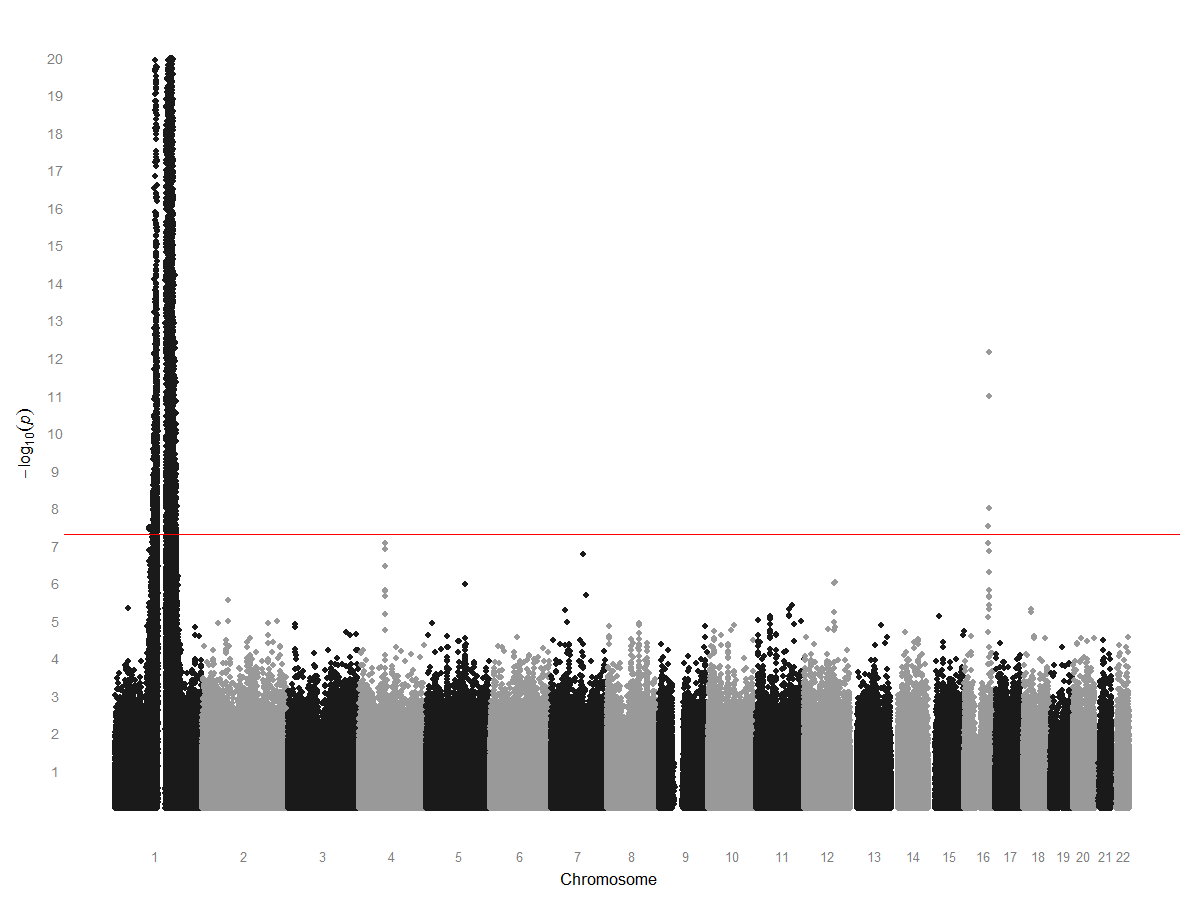


NEUTROPHILS


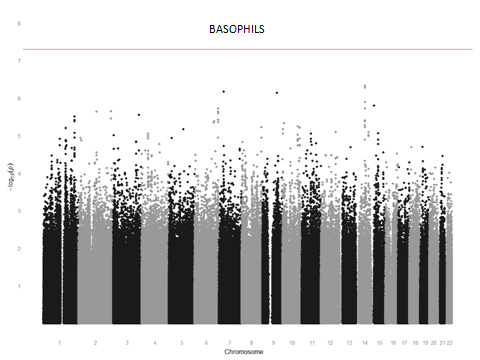

Supplement: Figure S2 — Manhattan of plots for neutrophil, lymphocyte, monocyte, eosinophil, and basophil counts. (DOC) [file pgen.1002108.s002.doc]
