# Supplementary material for: Genome-Wide Association Study of White Blood Cell Count in 16,388 African Americans: the Continental Origins and Genetic Epidemiology Network (COGENT)
Source: PLoS Genet. 2011 Jun 30;7(6):e1002108. doi: 10.1371/journal.pgen.1002108 (PMC3128101; doi:10.1371/journal.pgen.1002108)
Supplement: Figure S5 — Linkage disequilibrium (LD) plots in the region of the chromosome 16 HYDIN locus. (DOC) [file pgen.1002108.s005.doc]

**Supplemental Figure 5**: Linkage disequilibrium (LD) plots in the region of the chromosome 16 *HYDIN* locus. Plots were generated using HaploView using LD patterns from Europeans (CEU) or Africans (YRI).
