# Supplementary material for: Genome-Wide Association Study of White Blood Cell Count in 16,388 African Americans: the Continental Origins and Genetic Epidemiology Network (COGENT)
Source: PLoS Genet. 2011 Jun 30;7(6):e1002108. doi: 10.1371/journal.pgen.1002108 (PMC3128101; doi:10.1371/journal.pgen.1002108)
Supplement: Table S1 — Summary of SNP information and genomic inflation factors for all association analyses performed in this study. (DOC) [file pgen.1002108.s006.doc]

**Supplemental Table 1. Summary of SNP information and genomic inflation factors for all association analyses performed in this study**

| Phenotypes |  | ARIC | CARDIA | GeneSTAR | Health ABC | HANDLS | JHS | WHI | Meta-analysis |
| --- | --- | --- | --- | --- | --- | --- | --- | --- | --- |
| Sample size |  | 2,664 | 943 | 934 | 898 | 862 | 1,992 | 8,095 | 16,388 |
| Genotype platform |  | Affy 6.0 | Affy 6.0 | Illumina 1Mv1_C | Illumina 1M-Duo | Illumina 1M | Affy 6.0 | Affy 6.0 |  |
| # Typed SNPs*  # Total SNPs* |  | 796,384  2,747,295 | 839,912  2,765,482 | 968,251  2,418,799 | 914,493  2,959,148 | 907,763  2,835,329 | 868,969  2,773,094 | 854,981  2,426,484 | 2,489,215 |
| Genomic Control Lambda** | Total WBC (minus chr 1)  Neutrophils (minus chr 1)  Lymphocytes  Monocytes  Basophils  Eosinophils | 1.06 (1.04)  1.07 (1.05)  1.01  1.03  1.00  1.02 | 1.02 (1.01)  1.03 (1.02)  0.99  1.02  0.99  1.00 | 1.04 (1.02)  1.04 (1.04)  1.03  1.03  NA  NA | 1.03 (1.01)  1.00 (0.99)  0.97  0.97  1.02  1.01 | 1.01 (0.98)  1.01 (1.01)  0.99  0.99  0.99  1.01 | 1.11 (1.08)  1.10 (1.07)  1.08  1.10  1.04  1.06 | 1.09 (1.05)  NA (NA)  NA  NA  NA  NA | 1.05 (0.98)  1.05 (0.98)  1.01  1.01  0.98  1.01 |
|  |  |  |  |  |  |  |  |  |  |

*The numbers of genotyped and total SNPs include those available for final analysis, after QC filtering based on genotyping success rates, missing data, minor allele frequency, and imputation quality.

**For total WBC and neutrophil counts, the genomic control lambdas were calculated with (without) chromosome 1 markers.
