# Supplementary material for: Genome-Wide Association Study of White Blood Cell Count in 16,388 African Americans: the Continental Origins and Genetic Epidemiology Network (COGENT)
Source: PLoS Genet. 2011 Jun 30;7(6):e1002108. doi: 10.1371/journal.pgen.1002108 (PMC3128101; doi:10.1371/journal.pgen.1002108)
Supplement: Table S9 — Locus-specific ancestry-adjusted analyses of WBC–associated loci. (DOC) [file pgen.1002108.s014.doc]

Supplemental Table 9. Association of new and previously reported WBC loci, according to local ancestry

|  |  |  |  | SNP unadjusted* |  | SNP adjusted for Local Ancestry |  | SNP effect in those with 2 estimated locus-specific African chromosomes |  | SNP effect in those with at least one locus-specific European chromosome |
| --- | --- | --- | --- | --- | --- | --- | --- | --- | --- | --- |
| SNP | Chr | Gene | Phenotype | Beta [95% CI]  (P-value) | N | Beta [95% CI]  (P-value) | N | Beta [95% CI]  (P-value) | N | Beta [95% CI]  (P-value) |
| rs2814778 | 1 | *DARC* | WBC | 0.284 [0.273, 0.295] (p<0.0001) | 13385 | 0.250 [0.182, 0.318] (p<0.0001) | 8647 | 0.148 [0.012, 0.284] (p=0.03) | 4738 | 0.163 [0.044, 0.283] (0.007) |
| rs9131 | 4 | *CXCL2* | WBC | -0.023 [-0.032, -0.014] (p<0.0001) | 13996 | -0.020 [-0.030, -0.011] (p<0.0001) | 8505 | -0.024 [-0.037, -0.011]  (p<0.0001) | 5491 | -0.016 [-0.029, -0.002]  (p=0.021) |
| rs445 | 7 | *CDK6* | WBC | 0.015 [0.004, 0.025] (p=0.006) | 13597 | 0.015 [0.005, 0.026] (p=0.004) | 8537 | 0.008 [-0.005, 0.020] (p=0.22) | 5060 | 0.030 [0.011, 0.049] (p=0.002) |
| rs4065321 | 17 | *PSMD3* | WBC | 0.013 [0.005, 0.020] (p=0.001) | 13573 | 0.012 [0.004, 0.019] (p=0.002) | 8496 | 0.008 [-0.002, 0.018] (p=0.10) | 5077 | 0.017 [0.005, 0.030] (p=0.005) |
| rs4895441 | 6 | *HSB1L-MYB* | WBC | 0.003 [-0.009, 0.015] (p=0.63) | 13598 | 0.006 [-0.007, 0.018] (p=0.37) | 8633 | 0.016 [-0.012, 0.044] (0.25) | 4965 | -0.004 [-0.033, 0.025] (0.78) |
| rs3094212 | 6 | *PSORS1C1* | WBC | -0.004 [-0.012, 0.004] (p=0.32) | 13546 | -0.002 [-0.010, 0.005] (p=0.55) | 8292 | 0.004 [ -0.007, 0.014] (p=0.51) | 5254 | -0.011 [-0.025, 0.004] (p=0.16) |
| rs12313946  (rs6581830) | 12 | *RAP1B* | WBC | -0.006 [-0.014, 0.002]  (p=0.016) | 13598 | -0.005 [-0.013, 0.004]  (p=0.27) | 8754 | -0.003 [-0.014, 0.007] (p=0.53) | 4844 | -0.005 [-0.019, 0.008]  (p=0.42) |
| rs2072910 | 20 | *PCLB4* | Neutrophils | 0.003 [-0.011, 0.018] (p=0.63) | 5355 | 0.003 [-0.012, 0.018] (p=0.69) | 3778 | -0.001 [-0.019, 0.017] (p=0.92) | 1757 | 0.017 [-0.011, 0.046] (p=0.24) |
| rs4794822 | 17 | *PSMD3* | Neutrophils | -0.003 [-0.018, 0.012] (p=0.71) | 5535 | -0.002 [-0.017, 0.013] (p=0.77) | 3720 | -0.004 [-0.023, 0.015] (p=0.71) | 1815 | 0.003 [-0.022, 0.028] (p=0.81) |
| rs1420101 | 2 | *IL1RL1* | Eosinophils | -0.001 [-0.006, 0.003] (p=0.58) | 5402 | 0.003 [-0.009, 0.002] (p=0.25) | 3573 | -0.000 [-0.006, 0.005] (p=0.91) | 1829 | 0.001 [-0.007, 0.009] (p=0.80) |
| rs12619285 | 2 | *IKZF2* | Eosinophils | -0.004 [-0.009, 0.002]  (p=0.17) | 5382 | -0.004 [-0.009, 0.002]  (p=0.17) | 3631 | -0.004 [-0.009, 0.001]  (p=0.15) | 1751 | -0.003 [-0.015, 0.009] (p=0.61) |
| rs4857855 | 3 | *GATA2* | Eosinophils | 0.008 [-0.002, 0.019] (p=0.12) | 5402 | 0.008 [-0.002, 0.019] (p=0.12) | 3603 | 0.009 [-0.001, 0.018] (p=0.18) | 1799 | 0.008 [-0.003, 0.020] (p=0.15) |
| rs4143832 | 5 | *IL5* | Eosinophils | -0.000 [-0.004, 0.004] (p=0.95) | 5330 | -0.000 [-0.005, 0.004] (p=0.88) | 3482 | 0.002 [-0.004, 0.007] (p=0.53) | 1848 | 0.002 [-0.006, 0.009] (p=0.65) |
| rs3184504 | 12 | *SH2B3* | Eosinophils | -0.005 [-0.013, 0.003] (p=0.19) | 5402 | -0.004 [-0.011, 0.003] (p=0.31) | 3654 | 0.002 [-0.010, 0.014] (p=0.74) | 1748 | -0.010 [-0.021, 0.002] (p=0.09) |

*The baseline or "unadjusted model" is linear regression of ln-WBC phenotype on age, sex, center, PC1-PC10, and SNP genotype. The SNP genotype was coded dominantly for DARC and additively for all other loci.
