# Supplementary material for: Genome-Wide Association Study of White Blood Cell Count in 16,388 African Americans: the Continental Origins and Genetic Epidemiology Network (COGENT)
Source: PLoS Genet. 2011 Jun 30;7(6):e1002108. doi: 10.1371/journal.pgen.1002108 (PMC3128101; doi:10.1371/journal.pgen.1002108)
Supplement: Table S10 — Heritability estimates of WBC phenotypes. (DOC) [file pgen.1002108.s015.doc]

Table S10: African-American heritability estimates of WBC phenotypes based on 236 pedigrees from the GeneSTAR study*

| Trait | n | Heritability | SE | P-value |
| --- | --- | --- | --- | --- |
| WBC (unadjusted) | 934 | 0.483696 | 0.083042 | < 1.00e-07 |
| WBC (age-sex adjusted) | 934 | 0.477166 | 0.083136 | < 1.00e-07 |
| Monocyte count (unadjusted) | 806 | 0.292269 | 0.092074 | 0.000751 |
| Monocyte count (age-sex adjusted) | 806 | 0.292145 | 0.091738 | 0.000725 |
| Lymphocyte count (unadjusted) | 840 | 0.377276 | 0.090925 | 1.67e-05 |
| Lymphocyte count (age-sex adjusted) | 840 | 0.358722 | 0.091102 | 4.12e-05 |
| Granulocyte count (unadjusted) | 813 | 0.488019 | 0.090989 | < 1.00e-07 |
| Granulocyte count (age-sex adjusted) | 813 | 0.483428 | 0.090316 | < 1.00e-07 |

*All traits were ln-transformed.
