# Supplementary material for: Genome-Wide Association Study of White Blood Cell Count in 16,388 African Americans: the Continental Origins and Genetic Epidemiology Network (COGENT)
Source: PLoS Genet. 2011 Jun 30;7(6):e1002108. doi: 10.1371/journal.pgen.1002108 (PMC3128101; doi:10.1371/journal.pgen.1002108)
Supplement: Table S11 — Characteristics and distributions of traits in the study populations by the CHARGE Consortium. (DOC) [file pgen.1002108.s016.doc]

**Supplemental Table 11.** Characteristics and distributions of traits in the study populations by the CHARGE Consortium.

|  | Cohorts enrolled in CHARGE Consortium | | | | | | |
| --- | --- | --- | --- | --- | --- | --- | --- |
|  | **AGES** | **ARIC** | **Baltimore Longitudinal Study of Aging** | **Framingham Heart Study** | **Health ABC** | **InChianti** | **Rotterdam Study** |
| Total No. subjects | 3,217 | 4,846 | 337 | 3,909 | 1,075 | 1,014 | 5,111 |
| Age (mean ± SD) | 76.4 (5.5) | 54.3 (5.7) | 66.8 (13.9) | 35.9 (10.4) | 75.7 (2.8) | 68.1 (15.3) | 69.1 (9.0) |
| Female (%) | 58.0 | 53.2 | 48.7 | 51.2 | 47.1 | 57.0 | 59.5 |
| Total WBC (×10^3^/μL) | 6.01 (1.79) | 5.93 (1.40) | 5.44 (1.10) | 4.07 (0.23) | 6.17 (1.37) | 5.96 (1.26) | 6.49 (1.50) |
| Neutrophil (×10^3^/μL) | 3.51 (1.30) | 3.65 (1.12) | 3.15 (0.87) | N.A. | 3.66 (1.01) | 3.63 (1.02) | N.A. |
| Lymphocyte (×10^3^/μL) | 1.73 (0.94) | 1.81 (0.48) | 1.64 (0.45) | N.A. | 1.74 (0.58) | 1.83 (0.53) | 2.50 (0.78) |
| Monocyte (×10^3^/μL) | 0.54 (0.18) | 0.34 (0.14) | 0.41 (0.15) | N.A. | 0.53 (0.15) | 0.31 (0.09) | N.A. |
| Basophil (×10^3^/μL) | 0.029 (0.025) | 0.025 (0.033) | 0.012 (0.015) | N.A. | 0.060 (0.031) | 0.026 (0.019) | N.A. |
| Eosinophil (×10^3^/μL) | 0.207 (0.144) | 0.104 (0.103) | 0.174 (0.093) | N.A. | 0.173 (0.102) | 0.171 (0.091) | N.A. |

N.A., data not available.
