# Supplementary material for: Genome-Wide Association Study of White Blood Cell Count in 16,388 African Americans: the Continental Origins and Genetic Epidemiology Network (COGENT)
Source: PLoS Genet. 2011 Jun 30;7(6):e1002108. doi: 10.1371/journal.pgen.1002108 (PMC3128101; doi:10.1371/journal.pgen.1002108)
Supplement: Text S1 — Supplemental Materials and Methods. (DOC) [file pgen.1002108.s017.doc]

**SUPPLEMENTAL MATERIALS AND METHODS**

1. **COGENT AFRICAN AMERICAN STUDY PARTICIPANTS**
2. **Women's Health Initiative (WHI)-SHARe**

WHI is one of the largest (n=161,808) studies of women's health ever undertaken in the U.S. There are two major components of WHI: (1) a Clinical Trial (CT) that enrolled and randomized 68,132 women ages 50 – 79 into at least one of three placebo-control clinical trials (hormone therapy, dietary modification, and calcium/vitamin D); and (2) an Observational Study (OS) that enrolled 93,676 women of the same age range into a parallel prospective cohort study [1]. A diverse population including 26,045 (17%) women from minority groups were recruited from 1993-1998 at 40 clinical centers across the U.S. Of the CT and OS minority participants enrolled in WHI, 12,157 (including 8,515 self identified African American and 3,642 self identified Hispanic subjects) who had consented to genetic research were eligible for the WHI SHARe GWAS project. DNA was extracted by the Specimen Processing Laboratory at the Fred Hutchinson Cancer research Center (FHCRC) using specimens that were collected at the time of enrollment. Specimens were stored at -80°C. Blood samples for WBC analyses were collected at baseline.

**2. Atherosclerosis Risk in Communities Study (ARIC)**
The ARIC study is a prospective population-based study of atherosclerosis and cardiovascular diseases in 15,792 men and women, including 11,478 non-Hispanic whites and 4,314 African Americans, drawn from 4 U.S. communities (suburban Minneapolis, Minnesota; Washington County, Maryland; Forsyth County, North Carolina, and Jackson, Mississippi) [2]. Only self-reported African-American participants are included in this analysis. Participants were between age 45 and 64 years at their baseline examination in 1987-1989 when blood was drawn for DNA extraction and participants consented to genetic testing. Blood for white blood count analysis was drawn at the baseline exam. After taking into account availability of adequate amounts of high quality DNA, appropriate informed consent and genotyping quality control and assurance procedures, genotype data were available on 2,989 African-American individuals.

**3. Coronary Artery Risk Development in Young Adults (CARDIA)**

The CARDIA study is a prospective, multi-center investigation of the natural history and etiology of cardiovascular disease in African Americans and whites 18-30 years of age at the time of initial examination [3]. The CARDIA sample was recruited at random during 1985-86 primarily from geographically based populations in Birmingham AL, Chicago IL, and Minneapolis MN and, in Oakland, CA, from the membership of the Kaiser-Permanente Health Plan. The initial examination included 5,115 participants selectively recruited to represent proportionate racial, gender, age, and education groups from each of the four communities. Each participant’s age, race, and sex were self-reported during the recruitment phase and verified during the baseline clinic visit. Details of the study design and procedures for data collection have been published. From the time of initiation of the study in 1985-1986 (baseline examination), five follow-up examinations have been conducted at years 2, 5, 7, 10, 15, and 20. DNA extraction for genetic studies was performed at the Y10 examination. Blood for white blood count analysis was drawn at the baseline exam. After taking into account availability of adequate amounts of high quality DNA, appropriate informed consent and genotyping quality control and assurance procedures, genotype data were available on 955 African-American individuals.

**4. Jackson Heart Study (JHS)**

The Jackson Heart Study (JHS) is a prospective population-based study to seek the causes of the high prevalence of common complex diseases among African Americans in the Jackson, Mississippi metropolitan area, including cardiovascular disease, type-2 diabetes, obesity, chronic kidney disease, and stroke [4]. During the baseline examination period (2000-2004) 5,301 self-identified African Americans were recruited from four sources, including (1) randomly sampled households from a commercial listing; (2) ARIC participants; (3) a structured volunteer sample that was designed to mirror the eligible population; and (4) a nested family cohort. Unrelated participants were between 35 and 84 years old, and members of the family cohort were ≥ 21 years old when consent for genetic testing was obtained and blood was drawn for DNA extraction. Based on DNA availability, appropriate informed consent, and genotyping results that met quality control procedures, genotype data were available for 3,030 individuals, including 885 who are also ARIC participants. In the current study, JHS participants who were also enrolled in the ARIC study were analyzed with the ARIC dataset – for this reason, the JHS dataset analyzed here is defined as 2,145 individuals.

**5. Health, Aging, and Body Composition (Health ABC) Study**

The Health ABC study is a prospective cohort study investigating the associations between body composition, weight-related health conditions, and incident functional limitation in older adults. Health ABC enrolled well-functioning, community-dwelling African-American (n=1,281) and white (n=1,794) men and women aged 70-79 years between April 1997 and June 1998. Participants were recruited from a random sample of all Medicare eligible residents in the Pittsburgh, PA, and Memphis, TN, metropolitan areas. Eligibility requirements included no difficulty with activities of daily living, walking a quarter of a mile, or climbing 10 steps without resting. Participants have undergone annual exams and semi-annual phone interviews. Blood for WBC analysis was drawn at the baseline examination.

**6. The Healthy Aging in Neighborhoods of Diversity across the Life Span Study (HANDLS)**

HANDLS is an interdisciplinary, community-based, prospective longitudinal epidemiologic study examining the influences of race and socioeconomic status (SES) on the development of age-related health disparities among socioeconomically diverse African Americans and whites in Baltimore. The study domains include: nutrition, cognition, biologic biomarkers, body composition and bone quality, psychophysiology, physical function and performance, sociodemographics, psychosocial, neighborhood environment and cardiovascular disease. A total of 3,722 participants were recruited from Baltimore, MD with mean age 47.7 (range 30-64) years, including 2,200 African Americans (59%) and 1,522 whites (41%); 41% reported household incomes below the 125% poverty delimiter. The genetic component of HANDLS includes 1024 African American participants with genotyping data that has passed rigorous quality control procedures described in the following section.

**7. GeneSTAR**

GeneSTAR (The Johns Hopkins Genetic Study of Atherosclerosis Risk) is a longitudinal family based epidemiologic study examining  inflammatory, platelet, lipoprotein, blood pressure, metabolic, and vascular property determinants of incident atherosclerotic coronary heart disease and attendant co-morbidity (stroke and peripheral vascular disease) among apparently healthy first degree adult relatives of hospitalized probands with documented premature coronary disease events prior to 60 years of age.  Between 1990 and 2006, GeneSTAR enrolled 1418 asymptomatic, apparently healthy young African American siblings (<60 years of age) and offspring (21-59 years of age) of both the proband and his/her siblings, as well as co-parents of the offspring; of these, 934 had both genotype data which passed strict quality control measures and WBC phenotype data. African American participants represented 327 different families. Additionally 2,057 whites were enrolled in the same period. The current study is limited to African Americans.

**B. GENOTYPING AND QUALITY CONTROL IN COGENT DISCOVERY COHORTS**

**1. Candidate Gene Association Resource (CARe) Cohorts.** ARIC, CARDIA, and JHS samples were genotyped at the Broad Institute using the Affymetrix Genome-Wide Human SNP Array 6.0 (Affy6.0) according to the manufacturer’s recommendations, as part of the NHLBI Candidate Gene Association Resource (CARe) project. Genotyping and quality control procedures have been described in detail [5]. The Affy6.0 genotyping platform interrogates simultaneously 1.8 million markers for genetic variation (906,600 SNPs and 946,000 copy number variation probes). Several quality control (QC) procedures were performed on the genotype data, separately for each cohort. Quantity of double stranded DNA was assessed using PicoGreen® (Molecular Probes, Oregon, USA). To confirm sample identity, genotype concordance was evaluated for 24 SNPs genotyped in the same DNA samples using both Sequenom iPLEX and Affy6.0. Genome-wide genotype data were used to estimate identity-by-descent (IBD) between all pairwise combinations of samples in order to identify sample duplicates, contaminated samples, and cryptic relationships. We also used IBS/IBD measures to confirm known pedigree data for JHS. SNPs and samples with an unusually high number of Mendel errors were excluded. Heterozygosity rates (in the form of inbreeding coefficients) were estimated to identify problematic DNA samples (poor DNA quality or contaminations). DNA samples with a genome-wide genotyping success rate <95%, duplicate discordance or sex mismatch, SNPs with genotyping success rate <90%, monomorphic SNPs, SNPs with minor allele frequency (MAF) <1%, and SNPs that map to several genomic locations were removed from the analyses. The Hardy-Weinberg equilibrium (HWE) test was performed for all SNPs, but SNPs were not excluded based uniquely on this criterion given the admixed nature of the cohorts genotyped. After applying all quality control filters, the following numbers of African-American participants were available for analysis: ARIC=2,830, CARDIA=949, and JHS=2,144.

**2. WHI.** Genotyping was done at Affymetrix Inc on the Affymetrix 6.0 array, using 2 ug DNA at a concentration of 100 ng/ul. 2% (238) additional samples were genotyped as blind duplicates. When needed multiple attempts were made to genotype samples. Approximately 1% of samples could not be genotyped (n=99). We first removed samples that had call rate below 95% (n=16), that were duplicates of subjects other than monozygotic twins (n=34), or that had a Y-chromosome (n=1). SNPs that were located on the Y chromosome or were Affymetrix QC probes (not intended for analysis) were excluded (n=3280). We also flagged SNPs that had call rates, calculated separately for African Americans and Hispanics, below 95% and concordance rates below 98%, leaving us 871,309 unflagged SNPs. We computed IBD coefficients between all pairs of 12,008 subjects using a random subset of 100,000 SNPs from autosomal chromosomes [6]. Based on these coefficients we identified pairs of parent-offspring (22 pairs and two trios), monozygotic twins (five pairs) and siblings (192 pairs and five trios). A more thorough confirmatory analysis using a pairwise kinship coefficient estimator [7] was performed separately for African Americans and Hispanics that validated these relationships and identified half-siblings (73 pairs). In most analyses we only included one of each pairs of relatives, typically the one with the largest call rate. We were left with 12,008 unique subjects (8,421 African Americans and 3,587 Hispanics), with an average call rate of 99.8% over the unflagged SNPs. We analyzed 188 pairs of blind duplicate samples. The overall concordance rate was 99.8% (range 94.5-100% over all samples, 98.3%-100% over samples with call rate >98%, 98.1-100%% over unflagged SNPs).

**3. Health ABC.** For Health ABC, genotyping was performed by the Center for Inherited Disease Research (CIDR) using the Illumina Human1M-Duo BeadChip system. Genomic DNA was extracted from buffy coat collected during the baseline exam using a PUREGENE® DNA Purification Kit. Samples were excluded from the dataset for reasons of sample failure, genotypic sex mismatch, and first-degree relative of an included individual based on genotype data.  Genotyping was successful for 1,151,215 SNPs in 1,139 African Americans. For African Americans, genotypes were available on 1,007,948 high quality SNPS for imputation based on a 1:1 mixture of the CEPH:Yoruba (YRI) reference panel (release 22, build 36).  A total of 3,021,329 SNPs in AAs are available for analysis.

**4. HANDLS**. Genotyping was focused on a subset of participants self-reporting as African American, and was performed at the Laboratory of Neurogenetics, National Institute on Aging, National Institutes of Health. 1024 participants were successfully genotyped to 907763 SNPS at the equivalent of Illumina 1M SNP coverage (709 samples using Illumina Human 1M and 1M duo arrays, the remainder using a combination of 550K, 370K, 510S and 240S to equate the million SNP level of coverage), passing inclusion criteria into the genetic component of the study. Initial inclusion criteria for genetic data in HANDLS includes concordance between self reported sex and sex estimated from X chromosome heterogeneity, > 95% call rate per participant (across all equivalent arrays), concordance between self-reported African ancestry and ancestry confirmed by analyses of genotyped SNPs, and no cryptic relatedness to any other samples at a level of proportional sharing of genotypes > 15% (effectively excluding 1st cousins and closer relatives from the set of participants used in analyses). In addition, SNPs were filtered for HWE p-value < 1x 10^-7^, MAF < 0.01, and call rate < 95%. Basic genotype quality control and data management was conducted using PLINKv1.07. Cryptic relatedness was estimated via pairwise identity by descent analyses in PLINK [6] and confirmed using RELPAIR [8].

**5. GeneSTAR**

In GeneSTAR, SNP genotyping was performed at deCODE Genetics, Inc. using the Human 1Mv1_C array from Illumina, Inc. where 1,044,094 markers were released with an average call rate per sample of 99.65% and an overall missing data rate of 0.35%.  PLINK v1.06 [6] was used to detect and remove Mendelian errors. Hardy-Weinberg equilibrium (HWE) and minor allele frequency (MAF) for each SNP was tested in a defined set of independent subjects (n=326) representing the founders of the pedigrees. We detected no deviation from HWE for any of the SNPs at our threshold of 0.0005 (i.e. p= 0.05/number of SNPs tested). Admixture estimates were obtained using a subset of 18,982 SNPs from the GWAS array that were selected on the basis of low SNP correlation and high Fst optimal for the differentiation of Ceph, Yoruba, and Chinese + Japanese HapMap ([www.hapmap.org](http://www.hapmap.org/)) samples selected as ancestral reference populations. Using STRUCTURE (v2.2; <http://pritch.bsd.uchicago.edu/software>) the mean estimated Yoruba ancestry in the GeneSTAR African American participants was 79.29% (range 41.41% - 99.98%).

**C. PRINICIPAL COMPONENT ANALYSIS (PCA) AND ADJUSTMENT FOR POPULATION STRATIFICATION**

PCA was implemented in EIGENSTRAT [9] on cleaned African-American GWA genotype data using a subset of markers in common between the cohort samples and the reference panels. To identify axes of variation, we included global HapMap and/or HGDP populations (e.g., CEU, YRI, CHB+JPT, Native American) genotyped on the same platform as reference or seed samples. These reference populations were cleaned prior to PCA to remove population outliers. Examination of plots of the main principal components (PCs) for each cohort showed a high degree of correlation of the first PC with global European vs. African ancestry (*r*^2^ >0.90), as calculated independently using the population genetics software STRUCTURE or FRAPPE [10,11]. Participants were excluded if PC estimates for the first 4 PC were inconsistent with HapMap ASW samples or were PCA outliers (for example, 56 WHI, 8 ARIC, and 2 CARDIA subjects who were self-identified as African American, but who appeared to have less than 10% African ancestry). Study-specific criteria were used to determine the number of principal components used as covariates in regression analyses, ranging from 1 PC to 10 PC.

**D. GENOTYPE IMPUTATION**

Imputation in African-Americans was performed using MaCH 1.0.16, which requires phased reference haplotypes [12]. Individuals with pedigree relatedness or cryptic relatedness (pi_hat > 0.05) were filtered prior to imputation. SNPs with MAF ≥1%, call rate ≥97% and HWE *P* ≥10^-6^ were used for imputation. A combined CEU+YRI reference panel from HapMap phase 2 (release 22, build 36) was used [13]. A randomly selected subset of individuals from each cohort sample was used to generate recombination and error rate estimates. These rates were then used to estimate genotype dosages in all sampled individuals across the entire reference panel for over 2 million SNPs. Imputation results were filtered using a minimum imputation quality score, indicated by the RSQ_HAT estimate in MaCH of >0.5 and a MAF threshold of >1%.

In WHI, on a small test sample (2% of the markers on three chromosomes), the average R-squared was 0.88, with an allelic discordance rate of 2.3%. In the CARe cohorts, SNP imputation performance using the CEU+YRI panel was assessed by comparing imputed genotypes for SNPs genotyped on an independent genotyping platform. The allelic concordance rate was ~95.6%, calculated as 1 – 1/2*|imputed_dosage – chip_dosage|. This concordance rate is comparable to those calculated for individuals of African descent imputed with the HapMap 2 YRI individuals.

**E. MUTLI-ETHNIC REPLICATION COHORTS**

**1. CHARGE Consortium European Americans**

The European-American GWAS replication sample comprised 19,509 subjects from 7 CHARGE cohorts (AGES, ARIC, BLSA, FHS, Health ABC, InChianti and RS). Details of the CHARGE consortium including subject details and study designs, are described elsewhere [14] and are reported in an accompanying manuscript [15]. For the current analysis, WBC phenotypes were derived from data provided by fluorescence activated cell sorting technologies commonly employed in clinical and epidemiological studies to interrogate common hematological elements found in peripheral blood. Each study excluded all participants with any WBC measure outside of +/- 2 standard deviations from the mean value for that trait. The CHARGE cohort participant characteristics for the current total WBC count replication analysis are summarized in **Table S11**.
**2. WHI Hispanic Americans**

Our Hispanic-American replication sample was derived from the 3,642 self identified Hispanic subjects enrolled in the WHI-SHARe GWAS (see sections A1 and B2 under **Supplemental Methods** for details of subject selection, genotyping on the Affymetrix 6.0 platform, and quality control procedures). After applying exclusion criteria similar to those described for the WHI-SHARe African American participants, total WBC count phenotype and genotype data were available for 3,551 Hispanic Americans. The mean (S.D.) age was 60.2 (6.7) years and 100% were female. Genotype imputation has yet to be performed for the WHI-SHARe Hispanic American sample due to the lack of an appropriate Hispanic or Native American reference panel.

**3. RIKEN Japanese**

The Japanese replication sample consists of 14,767 participants with total WBC count, originally obtained as part of the BioBank Japan GWAS project [16]. The mean age was 62.3 ± 10.5 years and 34.5% were female. For the current analysis, WBC count was derived from medical records. Genotyping was performed using Illumina HumanHap610-Quad Genotyping BeadChip or Illumina HumanHap550v3 Genotyping BeadChip. Subjects with call rates < 0.98, closely related subjects based on the identity-by-descent (IBD), and subjects who were determined to be of non-Japanese origin by either self-report or by PCA were excluded from analysis. SNPs with MAF < 0.01 or with an exact P-value of the Hardy-Weinberg equilibrium test < 1.0 × 10^-7^ were excluded. Genotype imputation was performed using MACH 1.0 and genotype data from Phase II HapMap JPT and CHB individuals (release 24) as reference panel. Quality control filters of MAF ≥ 0.01 and *Rsq* values ≥ 0.7 were applied for the imputed SNPs.

**REFERENCES**

1. Design of the Women's Health Initiative clinical trial and observational study (1998) The Women's Health Initiative Study Group. Control Clin Trials. 19:61-109.
2. The Atherosclerosis Risk in Communities (ARIC) Study: design and objectives (1989) The ARIC investigators. Am J Epidemiol, 129: 687-702.
3. Friedman GD, Cutter GR, Donahue RP, Hughes GH, Hulley SB, et al (1988) CARDIA: study design, recruitment, and some characteristics of the examined subjects. J Clin Epidemiol 41:1105-1116.
4. Taylor HA, Jr., Wilson, JG, Jones DW, Sarpong, DF, Srinivasan A, et al (2005) Toward resolution of cardiovascular health disparities in African Americans: design and methods of the Jackson Heart Study. Ethn Dis 15: S6-4-17.
5. Lettre G, Palmer CD, Young T,Ejebe KG, Allayee H (2010) Genome-wide association study of coronary heart disease and its risk factors in 8,090 African Americans: The NHLBI CARe Project, PLoS Genet. In Press.
6. Purcell S, Neale B, Todd-Brown K, Thomas L, Ferreira MA, et al (2007) PLINK: a tool set for whole-genome association and population-based linkage analyses*.* Am J Hum Genet 81: 559-575.
7. Thornton T, McPeek MS. ROADTRIPS: case-control association testing with partially or completely unknown population and pedigree structure (2010) Am J Hum Genet 86:172-84.
8. Epstein MP, Duren WL, Boehnke M. (2000) [Improved inference of relationships for pairs of individuals](http://csg.sph.umich.edu/boehnke/relpair.pdf). Amer J Hum Genet 67:1219-1231.
9. Price AL, Patterson NJ, Plenge RM, Weinblatt ME, Shadick NA et al (2006) Principal components analysis corrects for stratification in genome-wide association studies. Nat Genet, 38, 904-909.
10. Pritchard JK, Stephens M, Donnelly P (2000) Inference of population structure using multilocus genotype data. Genetics 155, 945-59.
11. Tang H, Peng J, Wang P, Risch NJ (2005) Estimation of individual admixture: analytical and study design considerations. Genet Epidemiol. 28:289-301.
12. Li Y, Willer CJ, Ding J, Scheet P, Abecasis GR (2010) MaCH: using sequence and genotype data to estimate haplotypes and unobserved genotypes. Genet Epidemiol 34:816-834.
13. Huang L, Li Y, Singleton AB, Hardy JA, Abecasis G, Rosenberg NA, Scheet P (2009) Genotype-imputation accuracy across worldwide human populations. Am J Hum Genet 84:235-250.
14. Psaty BM, O'Donnell CJ, Gudnason V, Lunetta KL, Folsom AR et al. (2009) Cohorts for Heart and Aging Research in Genomic Epidemiology (CHARGE) Consortium: Design of prospective meta-analyses of genome-wide association studies from 5 cohorts. Circ Cardiovasc Genet 2: 73-80.
15. Nalls MA, Couper DJ, Tanaka T, Van Rooij FJA, Chen MH et al. (2010) Multiple loci are associated with white blood cell phenotypes PLos Genetics (submitted)
16. Kamatani Y, Matsuda K, Okada Y, Kubo M, Hosono N, et al (2010) Genome-wide association study of hematological and biochemical traits in a Japanese population. Nat Genet 42:210-215.

**ACKNOWLEDGEMENTS**

The authors acknowledge the essential role of the Cohorts for Heart and Aging Research in Genome Epidemiology (CHARGE) Consortium in development and support of this manuscript. CHARGE members include the Rotterdam Study (RS), Framingham Heart Study (FHS), Cardiovascular Health Study (CHS), the NHLBI’s Atherosclerosis Risk in Communities (ARIC) Study, and the NIA’s Iceland Age, Gene/Environment Susceptibility (AGES) Study. The collaboration of studies such as the Health Aging and Body Composition Study (Health ABC), the Baltimore Longitudinal Study of Aging (BLSA), the Invecchiare in Chianti Study (InChianti), and the Heart and Vascular Health Study (HVH) also played a vital role.

This research was made possible by NIA/NIH contract AG000932‐02 (2009) Characterization of Normal Genomic Variability. This study utilized the high‐performance computational capabilities of the Biowulf Linux cluster at the National Institutes of Health, Bethesda, MD [http://biowulf.nih.gov].

The Age, Gene/Environment Susceptibility Reykjavik Study is funded by NIH contract N01‐AG‐12100, the NIA Intramural Research Program, Hjartavernd (the Icelandic Heart Association) and the Althingi (the Icelandic Parliament).

The Atherosclerosis Risk in Communities Study is carried out as a collaborative study supported by National Heart, Lung, and Blood Institute contracts N01‐HC‐55015, N01‐HC‐55016, N01‐HC‐ 55018, N01‐HC‐55019, N01‐HC‐55020, N01‐HC‐55021, N01‐HC‐55022, and grants R01HL087641, R01HL59367 and R01HL086694; National Human Genome Research Institute contract U01HG004402; and National Institutes of Health contract HHSN268200625226C. The authors thank the staff and participants of the ARIC study for their important contributions. Infrastructure was partly supported by Grant Number UL1RR025005, a component of the National Institutes of Health and NIH Roadmap for Medical Research.

The National Heart, Lung, and Blood Institute's Framingham Heart Study is a joint project of the National Institutes of Health and Boston University School of Medicine and was supported bythe National Heart, Lung, and Blood Institute's Framingham Heart Study (contract No. N01‐HC‐25195) and its contract with Affymetrix, Inc. for genotyping services (contract No. N02‐HL‐6‐4278). Analyses reflect the efforts and resource development from the Framingham Heart Study investigators participating in the SNP Health Association Resource (SHARe) project. A portion of this research was conducted using the Linux Cluster for Genetic Analysis (LinGA‐II) funded by the Robert Dawson Evans Endowment of the Department of Medicine at Boston University School of Medicine and Boston Medical Center.

The Health ABC Study was supported in part by the Intramural Research Program of the NIH, National Institute on Aging, NIA contracts N01AG62101, N01AG62103, and N01AG62106. The genome‐wide association study was funded by NIA grant 1R01AG032098‐01A1 to Wake Forest University Health Sciences and genotyping services were provided by the Center for Inherited Disease Research (CIDR). CIDR is fully funded through a federal contract from the National Institutes of Health to The Johns Hopkins University, contract number HHSN268200782096C.

The InChianti Study was supported as a "targeted project" (ICS 110.1RS97.71) by the Italian Ministry of Health, by the U.S. National Institute on Aging (Contracts N01‐AG‐916413, N01‐AG‐821336, 263 MD 9164 13, and 263 MD 821336) and in part by the Intramural Research Program, National Institute on Aging, National Institutes of Health, USA.

Rotterdam Study GWAS database of the Rotterdam Study was funded through the Netherlands Organization of Scientific Research NWO (nr. 175.010.2005.011, 911.03.012) and the Research Institute for Diseases in the Elderly (RIDE). This study was supported by the Netherlands Genomics Initiative (NGI)/NWO project number 050 060 810 (Netherlands Consortium for Healthy Ageing). We thank Dr Michael Moorhouse, Pascal Arp, Mila Jhamai, Marijn Verkerk and Sander Bervoets for their help in creating the genetic database. We thank the laboratory technicians Jeannette M Vergeer ‐ Drop, Bernadette H M van Ast ‐ Copier, Andy A L J van Oosterhout, Sue Ellen Mauricia, Andrea J M Vermeij ‐ Verdoold, Els Halbmeijer ‐ van der Plas, Debby M S Lont, and Hasna Kariouh for their help in phenotype assessment. The Rotterdam Study is supported by the Erasmus Medical Center and Erasmus University, Rotterdam; the Netherlands organization for scientific research (NWO), the Netherlands Organization for the Health Research and Development (ZonMw), the Research Institute for Diseases in the Elderly (RIDE), the Netherlands Heart Foundation, the Ministry of Education, Culture and Science, the Ministry of Health, Welfare and Sports, the European Commission (DG XII), and the Municipality of Rotterdam.

We would like to thank all the staff of the Laboratory for Statistical Analysis at RIKEN for their technical assistance. This study was supported by Ministry of Education, Culture, Sports, Science and Technology, Japan.
